# Advances in thermal physiology of diving marine mammals: The dual role of peripheral perfusion

**DOI:** 10.1080/23328940.2021.1988817

**Published:** 2021-12-18

**Authors:** Arina B. Favilla, Markus Horning, Daniel P. Costa

**Affiliations:** a Department of Ecology and Evolutionary Biology, University of California, Santa Cruz, CA, United States; b Wildlife Technology Frontiers, Seward, AK, United States

**Keywords:** Biologging, blood flow, blubber, heat flux, seals, thermoregulation

## Abstract

The ability to maintain a high core body temperature is a defining characteristic of all mammals, yet their diverse habitats present disparate thermal challenges that have led to specialized adaptations. Marine mammals inhabit a highly conductive environment. Their thermoregulatory capabilities far exceed our own despite having limited avenues of heat transfer. Additionally, marine mammals must balance their thermoregulatory demands with those associated with diving (i.e. oxygen conservation), both of which rely on cardiovascular adjustments. This review presents the progress and novel efforts in investigating marine mammal thermoregulation, with a particular focus on the role of peripheral perfusion. Early studies in marine mammal thermal physiology were primarily performed in the laboratory and provided foundational knowledge through *in vivo* experiments and *ex vivo* measurements. However, the ecological relevance of these findings remains unknown because comparable efforts on free-ranging animals have been limited. We demonstrate the utility of biologgers for studying their thermal adaptations in the context in which they evolved. Our preliminary results from freely diving northern elephant seals (*Mirounga angustirostris*) reveal blubber’s dynamic nature and the complex interaction between thermoregulation and the dive response due to the dual role of peripheral perfusion. Further exploring the potential use of biologgers for measuring physiological variables relevant to thermal physiology in other marine mammal species will enhance our understanding of the relative importance of morphology, physiology, and behavior for thermoregulation and overall homeostasis.

## Introduction

Few endothermic organisms live at ambient temperatures at or near body temperature. The marine environment is particularly challenging for thermoregulation due to the high thermal conductivity of water [1]. Marine endotherms must maintain thermal balance while immersed in a medium that conducts heat roughly 25 times faster than air [2]. Additionally, the marine environment provides limited thermal refugia except perhaps in the vertical dimension or when hauling out. This thermally challenging environment has provided intense evolutionary pressure as endothermic tetrapods transitioned from terrestrial to aquatic living on multiple occasions [3,4]. While the underlying physiological mechanisms of marine mammals resemble those of humans, their thermoregulatory capabilities far exceed our own.

### Technological innovations enable physio-logging of marine mammals

**Figure 1.**
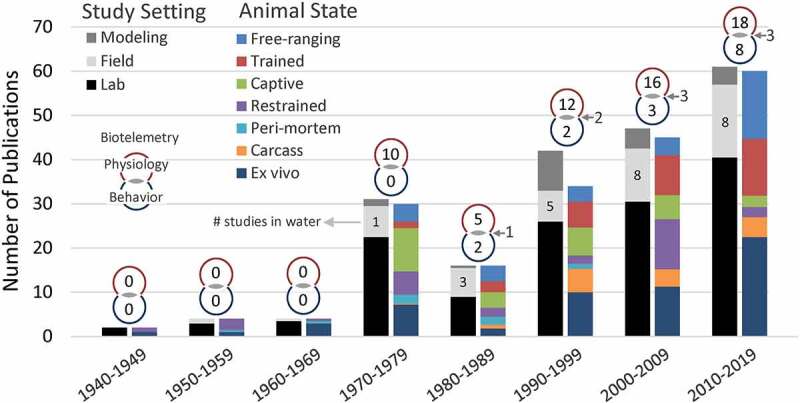
Number of publications (total = 207) by decade from 1940 to 2019 relevant to the thermal physiology of marine mammals. Refer to the supplementary file for the studies included. Studies are classified by setting (i.e. where it was performed) (left bar) and by the state or condition of the animal (right bar; modeling studies that did not make measurements on an animal were not included). Numbers inside the field bar (light gray) indicate how many of those field studies (if ≥1) involved animals in water (vs. land). Venn-diagrams on top of the bars indicate how many studies during that decade used biotelemetry for remote measurements of physiological variables (e.g. body temperature; top), or behavioral variables (e.g. diving behavior; bottom), or both. Biotelemetry is defined broadly here and includes remote sensing of physiologically relevant data (e.g. infrared thermography) and the use of data loggers (i.e. biologgers) to record continuous measurements on captive animals. Categories for study settings were defined as: lab (i.e. using an experimental approach in a captive and controlled setting), field (i.e. using wild animals in their natural setting), biophysical modeling (e.g. heat transfer models). Categories for animal state were defined as: *ex vivo* (i.e. excised tissue measurements), carcass (i.e. *in situ* measurements on a dead animal), peri-mortem (i.e. measurements taken at or near death), restrained (including sedated), captive (and unrestrained or freely-behaving given surrounding constraints), trained (i.e. accustomed to experimental protocol or performing a task on command), free-ranging (i.e. freely-behaving wild animals). Classification into these categories was inclusive (i.e. one study could be classified into multiple categories) and a study’s contribution to multiple categories was weighted evenly. Only research articles were included; book and encyclopedia chapters, theses, reviews, or comments were excluded. Papers that did not include any discussion of thermoregulation even if relevant parameters were measured (e.g. blubber lipid content, body composition, movement patterns in relation to sea surface temperature) were excluded.

Marine mammals display many extreme adaptations suitable for investigations aligned with the Krogh Principle .^1^ The increased thermal challenges often translate to greater signal-to-noise ratio that may facilitate mechanistic studies linking cause and effect, especially through the application of biologging devices. However, challenging logistics, limited animal access, and animal welfare issues may limit such studies in addition to technological challenges associated with high-pressure cycles in a corrosive environment [5]. As a result, most physiology research was carried out on dead (and/or excised tissue samples) or captive marine mammals. Field research, on the other hand, consisted primarily of observational studies of marine mammals on land (Figure 1).

**Figure 2.**
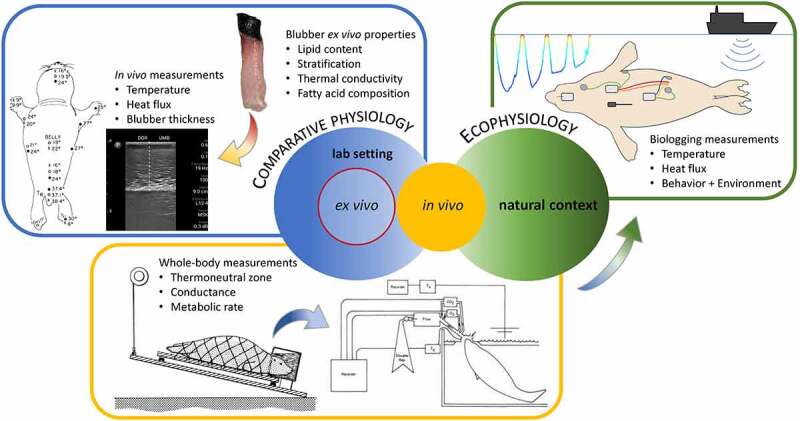
Graphical depiction of various approaches for understanding the thermal physiology of marine mammals. Both comparative physiology studies performed in the laboratory and ecophysiology studies (i.e. physiology studies conducted in the field where the ecological context is taken into consideration) have contributed to our understanding of marine mammal thermal physiology. However, most have relied heavily on species that are more readily accessible (e.g. seals). The blue box highlights how knowledge gained from *ex vivo* studies on blubber is relevant to understanding its function *in vivo* and its influence on other thermal measurements (e.g. skin temperature and heat flux). *In vivo* studies in the laboratory (yellow box) have provided insights into whole-body thermal dynamics using experimental methods that simulate reality to varying degrees (e.g. forced submersion experiments to trained dives). To understand the ecological relevance of their thermal limits, field studies (green box) using biologgers can record thermal responses during natural behavior and use these baseline measurements to compare those taken under disturbed conditions. Arrows depict efforts to translate findings using different approaches (*ex vivo* to *in vivo*, red to yellow) or in different contexts (lab setting to the natural context, blue to green). Skin and blubber biopsy sample from *Megaptera novaeangliae* and image (courtesy of L. Pallin) collected under scientific research permits NMFS 23095, ACA 2020–016, and UCSC IACUC Friea2004. Ultrasound image from *Mirounga angustirostris* collected under scientific research permits NMFS 21388 and UCSC IACUC Costad2009-1. Seal outline (© Jessica Kendall-Bar) was modified to depict instrumentation for research conducted under NMFS 19108 and 21388 and UCSC IACUC Costad2009-3. Seal outline depicting body temperature measurements adapted with permission from Miller & Irving 1975. Metabolism and temperature regulation in young harbor seals *Phoca vitulina richardi*. American Journal of Physiology 229: 506–511. Schematic of an experimental approach reprinted from Gallivan and Ronald, 1979. Temperature regulation in freely diving harp seals (*Phoca groenlandica*). Canadian Journal of Zoology, 57: 2256–2263. © Canadian Science Publishing. Figure depicting methods used for forced submersion experiments reprinted with permission from Zapol et al. 1979. Regional blood flow during simulated diving in the conscious Weddell seal. Journal of Applied Physiology, 47: 968:973. © American Physiological Society.

Recent biologging innovations are now revealing novel insights into the behavior and physiology of these difficult-to-study species (Figure 1). The application of biologging to study physiology remotely, a.k.a. physio-logging [6], has also enabled the extension of earlier, traditional controlled-access laboratory experiments (e.g.[7–11]) and modeling efforts (e.g. [12–15]) with field studies on free-ranging animals that are critical for determining the ecological relevance of findings derived from laboratory studies and modeling efforts (Figure 2). To demonstrate this, we present preliminary results from our research on the thermal physiology of freely diving elephant seals. By capturing fine-scale changes in their thermal responses while diving, our findings provide novel evidence of blubber’s dynamic nature and the complex interaction between thermoregulation and the dive response due to the dual role of peripheral perfusion.

## Marine mammals: Masters of endothermy in water

### An evolutionary perspective of their thermal adaptations

Extant marine tetrapods display diverse adaptations spanning morphology, physiology, and behavior that allow them to thrive in challenging thermal habitats. Species with longer evolutionary histories spend a greater proportion of time in the water. This has led to a convergence of morphological adaptations [3,16], including those pertinent to thermal physiology, such as a large size (i.e. small surface-area-to-volume ratio) and little to no hair [17]. Over time, the loss of hair or fur was possible due to an evolutionary transition to blubber as the primary insulation [18,19]. While mammals typically have subcutaneous fat, most marine mammals have a specialized blubber layer composed of adipose tissue reinforced by collagen and elastin [20,21]. The fully aquatic marine mammals, such as whales and dolphins (i.e. cetaceans), are the prime example of transitioning to this morphological adaptation [22]. They rely solely on blubber for insulation.

In contrast, amphibious marine mammals have retained fur in addition to having a blubber layer because of the unique challenge of dealing with the contrasting thermal properties of air and water. These species include seals and sea lions (i.e. pinnipeds) that forage at sea but must return to land to breed or molt. The relative importance of their insulation layers depends on the frequency and duration of time at sea. Some species can strategically partition their time between land and water to meet their thermal demands [23,24]. Other species are constrained by long seasonal periods in either environment. They must therefore be able to maintain thermal balance in both [25,26].

In water, blubber is advantageous due to its hydrodynamic qualities and greater functional resistance to pressure compared to fur. For this reason, deep divers do not rely on their fur for insulation while at depth. The pinnipeds’ broad spectrum of diving capabilities is reflected in their relative blubber thicknesses and fur densities [27]. These species provide interesting case studies for comparing and understanding the role of blubber in their thermal flexibility [28,29].

**Figure 3.**
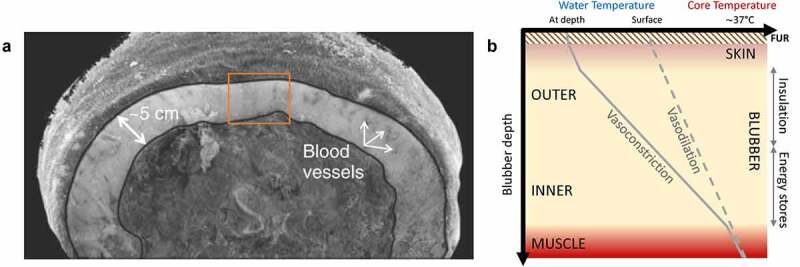
A cross-section from an adult Weddell seal (*Leptonychotes weddellii*) with blood vessels visible within the blubber layer. The orange box denotes the area represented in (b). Adapted with permission from Springer Nature: Marine Mammals by Randall W. Davis © 2019 (a). A conceptual figure depicting blubber’s various thermal states dependent on peripheral perfusion, and blubber’s functional roles due to its stratification. The temperature gradient across the blubber layer will vary based on the degree of peripheral perfusion during a dive. A larger gradient will generally occur at depth where lower water temperatures and peripheral vasoconstriction associated with the dive response lead to cooling of the periphery. The inner and outer blubber layers primarily serve different functions (energy store and insulation, respectively). This stratification is due to the relative proportional composition of fatty acids which confers different physical and biochemical properties (b).

What makes blubber the ideal insulator also makes it challenging for *in vivo* studies. Blubber is internal, which allows for blood to bypass this insulation when needed (Figure 3a). A complex microvasculature network exists within the skin and blubber of marine mammals [23,24,30–32]. Superficial vessels are associated with arteriovenous anastomoses. These structures allow fine-scale adjustment to blubber’s insulative and transmissive properties by either constricting or dilating to regulate peripheral perfusion. By effectively modifying their conductance, marine mammals control the characteristics of the thermal gradient (Figure 3b) and can maintain a high core body temperature while immersed in water [33,34]. Therefore, understanding the thermoregulatory strategies of diving marine mammals requires examining the physiological mechanisms that influence heat transfer at their periphery.

### Modes and spatial heterogeneity of heat transfer

Marine mammals experience fewer avenues of heat transfer in water: there is no evaporative heat loss, and radiation is minimal compared to other avenues of heat loss. Thus, conduction and convection, both of which depend on the temperature differential, are the principal mechanisms through which heat transfer occurs. The temperature gradient and the thickness of the blubber layer determine the conductive heat transfer through the blubber and ultimately how much core body heat reaches the skin. Convective heat transfer causes the heat at the skin’s surface to dissipate to the surrounding water. Unlike the thermal conductivity of blubber, which is a single readily measured property, the convective heat transfer coefficient depends on several factors, including fluid properties and type of fluid flow [35]. However, often neglected is the variability introduced by internal forced convection, which occurs due to active transfer of heat through the circulation of blood [36–38].

Many studies have referred to conduction through the blubber layer when both conductive and convective mechanisms were considered. To account for this, the blubber conductivity, which describes how readily heat transfers through a material, was adjusted using a correction (“perfusion”) factor (e.g. [15,39]). The values of marine mammal blubber conductivity are typically measured from excised blubber and genuinely reflect its intrinsic property. In live blubber, variable peripheral perfusion will contribute to heat transfer through active transport resulting in a different heat transfer rate than would be expected by conduction alone. Circulating blood thus sensibly changes the conductivity of the blubber layer [40–42]. Analyzing convective heat transfer is more complex than conduction. By incorporating a variable blubber conductivity relative to peripheral perfusion, the biophysical problem is simplified.

Kvadsheim and Folkow [38] measured the total heat transfer (conductive and convective) with heat flux sensors in harp seals (*Pagophilus groenlandicus*) resting in the water to determine the circulatory effects of heat transfer through the blubber. This was compared to the calculated conductive heat transfer based on the blubber temperature gradient measured with thermistors placed in the deep blubber and subcutaneously. Convective heat transfer was minimal in cold water, and heat loss was mainly from the trunk. An increasing proportion of convective heat loss from the flippers as water temperatures increased to ~23-24°C documented the role of flippers as heat dissipators when seals were heat-stressed. This has also been demonstrated for the flippers and flukes of cetaceans [41,43] and the hands and feet of humans [44]. The lack of insulation in these appendages and their large surface-area-to-volume ratio make these sites ideal as thermal windows, where rapid changes in heat transfer are induced through physiological mechanisms (i.e. regulating blood flow [44–46]).

**Figure 4.**
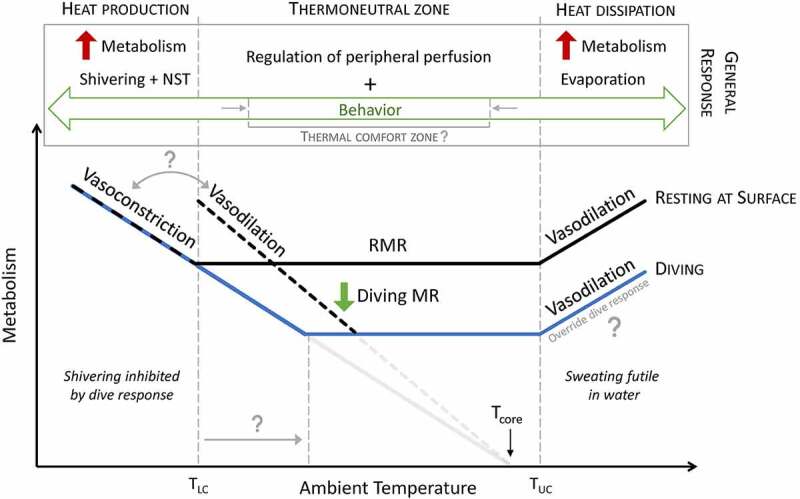
Schematic figure depicting the relationship between ambient temperature and metabolism (black line) and the general thermal responses within the thermoneutral zone, above the upper critical temperature (T_UC_), and below the lower critical temperature (T_LC_). For marine mammals in water, evaporative heat loss becomes futile, and diving imposes additional constraints on heat production due to the need to conserve oxygen. Therefore, marine mammals must compensate for these limited modes of heat exchange through vasomotor changes and behavior. Several questions remain to address how diving (and the associated metabolic response, blue line) modulates their thermal responses (represented by the question marks).

This experiment demonstrated how ambient temperatures influence vasomotor control in seals, albeit to varying degrees across the body, which aligns with the expansive literature on the effects of temperature on vasoconstriction and vasodilation in humans [47–51]. While equivalent mechanisms underlie peripheral perfusion, marine mammals may have greater thermal capabilities than humans, in part, due to a greater density and distribution of arteriovenous anastomoses. Arteriovenous anastomoses are primarily found in the glabrous skin of humans and play a significant role in regulating body temperature with minimal energy expenditure within the thermoneutral zone [46,50]. In contrast, marine mammals have arteriovenous anastomoses along their body surface – although some more than others [30,31] – which allows the well-insulated trunk to substantially contribute to total body heat flux [38,52,53]. The different thermal responses of glabrous (i.e. non-hairy) and hairy skin regions of humans are analogous to the different roles of flippers or fins and the body trunk of marine mammals [46]. However, the importance of skin temperature as a feedback signal in marine mammals is unclear especially since water dampens the variation in temperature across the skin compared to in air [41,53–60]. Given the limited modes of heat transfer in water and the additional constraints that diving imposes on regulating their body temperature (Figure 4), marine mammals are likely to rely on arteriovenous anastomoses and adjustments in peripheral perfusion as a primary mechanism for thermoregulation.

### Perspectives from the field of diving physiology

In addition to the challenges mentioned above faced by endotherms in the marine environment, free-ranging marine mammals must balance the physiological demands of thermoregulation with exercise and diving. The cardiovascular system is integral to the physiological responses associated with the dive response, exercise, and thermoregulation. These responses could either be in sync or conflict, requiring marine mammals to coordinate their activities or compromise their performance [27]. The dive response involves a suite of cardiovascular adjustments, including apnea, bradycardia, and peripheral vasoconstriction. Together they reduce blood flow and oxygen consumption of non-essential tissues to conserve their “on-board” oxygen supply for vital organs, such as the heart and brain [61]. Therefore, blood flow regulation is essential for the redistribution of oxygen and heat within the body. While endotherms are generally considered homeotherms, homeothermy may not be the most efficient strategy for marine mammals due to the physiological demands of diving [62]. Any regional or systemic drop in body temperature would reduce tissue’s metabolic requirements (i.e. oxygen consumption) due to the temperature dependence of metabolic energy production (quantified by the Q_10_ temperature coefficient). This would extend the aerobic dive limit by decreasing the rate at which on-board oxygen stores are depleted. Hypothermia-induced metabolic suppression and regional heterothermy have been posited as separate hypotheses to explain how marine mammals increase their aerobic dive capacity [63,64]. The role of hypothermia and peripheral cooling has been primarily discussed in the diving physiology literature [63–66]. Still, the thermoregulatory implications of these diving strategies have not been adequately investigated. By integrating and building on knowledge gained in the separate considerations of diving physiology and thermal physiology, current research is advancing our understanding of the complex interactions between the dive response and thermoregulation.

## Insights from *ex vivo* and *in vivo* lab studies

Unlike research in diving physiology that has embraced physio-logging to simultaneously record dive profiles along with physiological responses (e.g. heart rate and blood oxygen saturation) in freely diving species [65,67–74], progress in thermal physiology has heavily relied on *ex vivo* studies (e.g. [23,30,31,75–77]) or those on captive animals in the laboratory (e.g. [7,37,78–80]) (Figure 1). Similar to the differences between studying nude humans in the lab and humans in their routine states (e.g. clothed and working [29]), these laboratory findings demonstrated the maximum capabilities of marine mammals. Although these studies could not address whether or how often animals reached these maximum capabilities in their natural environments, they have, nonetheless, provided key insights that would not have been possible through field studies.

### Structure meets function in blubber

*Ex vivo* studies have enhanced our understanding of the role of blubber by investigating the physical (e.g. adipocyte morphology, thickness, thermal conductivity [81–85]) and biochemical (e.g. lipid content, fatty acid composition, and stratification [19,76,86–88]) properties of blubber (Figure 2) . These have shown that most marine mammals have stratified blubber layers. The outer layer primarily functions as insulation where lipids with lower melting points confer flexibility particularly at lower temperatures. In contrast, the composition of the inner layer makes it more readily metabolized and thus primarily serves as an energy store (Figure 3b). The relative importance of either function is also dependent on age and season [84,85,89], as well as species-specific thermal habitats and life-history strategies [32,75]. For example, blubber as an energy store may be more important for older animals during the breeding fast (especially for capital breeders) [90–92]. In contrast, blubber as an insulator may be more important for younger, smaller animals that have a greater body surface-area-to-volume ratio and/or are nutritionally dependent [7,84,93,94].

The tradeoffs between blubber’s dual role as insulation and an energy store are directly influenced by its relative thickness. Many studies using captive species have described the topographical distribution of the blubber layer and its significance [79,88,90,92,95–98]. In addition to seasonal and ontogenetic changes in blubber thickness, other changes in the blubber layer occur on much shorter time scales due to the physiological responses associated with diving, particularly the regulation of peripheral perfusion. Blubber is considered a dynamic insulator [99,100], and understanding its context-dependent roles can only be addressed *in vivo*.

### A dynamic insulator used as a buffer zone

**Figure 5.**
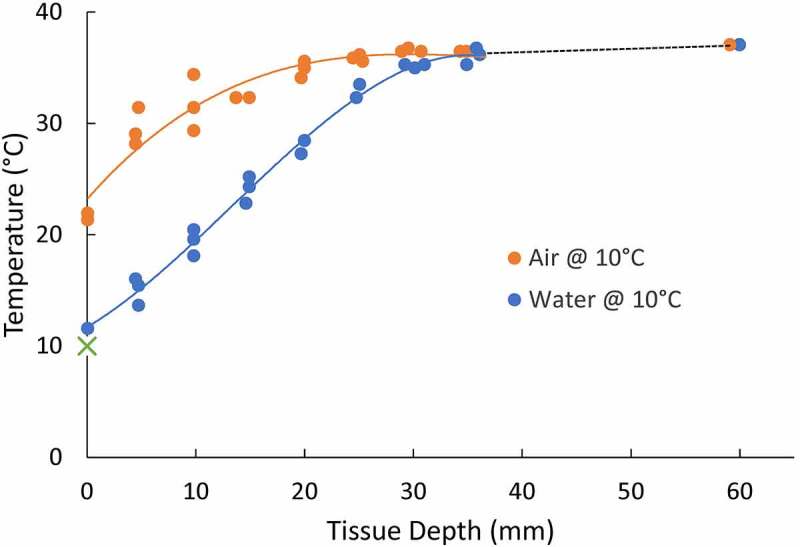
Tissue temperature gradients of a harbor seal (*Phoca vitulina*) in air (orange) and water (blue) at 10°C (green “x” on y-axis). Modified from Irving and Hart, 1957. The metabolism and insulation of seals as bare-skinned mammals in cold water. Canadian Journal of Zoology, 35: 497–511. © Canadian Science Publishing.

Many early studies investigated the thermoregulatory capabilities and whole-body thermal dynamics of marine mammals in laboratory settings (Figure 2). As early as the 1950s, experiments were performed *in vivo* to define a species’s lower critical temperatures and whether an elevated metabolic rate is required to maintain thermal balance in an aquatic environment [9,10,29,34,37,54,78,93,101–104]. Irving and Hart [34] found differences in the blubber gradient explained how seals exhibited similar metabolic rates in air and water of the same temperature despite the greater cooling effects of water (Figure 5). They concluded that peripheral cooling is essential to allow homeothermy in aquatic “bare-skinned” mammals. Such labile control of insulation through changes in vascular heat transfer would be advantageous over the static insulation provided by thick fur.‬‬‬‬‬‬‬‬‬‬‬‬‬‬‬‬‬‬‬‬‬‬‬‬‬‬‬‬‬‬‬‬‬‬‬‬‬‬‬‬‬‬‬‬‬‬‬‬‬‬‬‬‬‬‬‬‬‬‬‬‬‬‬‬‬‬

Fewer studies have investigated the upper critical temperatures of marine mammals while immersed. This is partly because they cannot respond to heat stress in water with evaporative cooling [12,105] (Figure 4). Without such an early warning indicator, there is the risk of surpassing their thermal tolerance limit, resulting in severe heat injury or death. Still, an experiment on captive bottlenose dolphins (*Tursiops truncatus*) resting in water examined how dolphins avoid hyperthermia when moving from cooler pelagic waters to warmer coastal waters [106]. Water temperature was progressively increased and then maintained above skin temperature to prevent heat loss from the body. Heat flux values showed the rate of heat gain decreasing over time. Simultaneously, rectal temperatures declined up to 1.3°C of baseline temperature. This reduction is surprising given the anticipated increase of 1.6°C based on basal metabolic heat production. These results imply that dolphins can redistribute core body heat to their periphery via increased peripheral blood flow. By using their blubber layer as a buffer zone to store heat, dolphins can achieve short-term heat tolerance (~1 hr) in coastal waters. Subcutaneous thermistors, alongside experiments investigating the phase change properties of blubber, could provide further evidence of blubber’s dynamic role and its influence on peripheral heat flow and metabolic rates.

While similar experiments with large cetaceans would be challenging, modeling indicates that the regulation of peripheral perfusion is critical for long-term thermal homeostasis. Models based solely on the insulative properties of the blubber layer suggest that large whales are over-insulated for their size and metabolism [15]. While blood flow and metabolism were estimated, models that included peripheral circulation provided reasonable predictions of *in situ* temperature profiles of freshly harpooned fin and sei whales in the mid-1900s [107]. These results suggest that whales must maintain some blood flow to the skin, even in the coldest waters they encounter, to maintain thermal balance. In warmer waters, the level of blood flow would increase exponentially.

These studies have provided key insights into the physiological responses to water temperature and thermal limits of species spanning three orders of magnitude in size and adapted to vastly different thermal habitats. However, the ecological relevance is limited since few marine mammals remain stationary in water for prolonged periods. Thus, an essential extension of these studies will require investigating how exercise and diving affect thermal dynamics.

### Thermal effects of exercise and diving

The paradoxical interaction between the physiological demands of diving and exercise has interested many researchers [108–112]. Studies have found that diving mammals seem to avoid the classic exercise response [1, 2,113] and are rather well-adapted for exercise while breath-holding. A reduction in heart rate associated with the dive response reduces metabolism and conserves oxygen but is contrary to the expected elevated heart rate during exercise [100,114,115]. Additionally, peripheral vasoconstriction reduces blood flow to the periphery during the dive, thus reducing oxygen supply to locomotory muscles [63]. To meet their exercising muscles’ oxygen demands during breath-hold dives, marine mammals swim efficiently and utilize their larger muscle myoglobin oxygen stores [116,117]. Studies have shown that the dive response is modulated by exercise [112,118–120]. Yet, whether the dive response is modulated by thermoregulatory demands and the associated consequences of exercise and diving on thermal homeostasis is unknown.

To investigate the thermal effects of exercise, the spatial patterns of heat flux in Steller sea lions (*Eumetopias jubatus*) were examined while stationary in the water, swimming naturally in a swim flume, and swimming under increased load [55]. Areas with less insulation (hips and shoulders) had the highest heat flux values compared to other more insulated areas (middle and axillary girths) regardless of the level of physical exertion. It was concluded that the exercising Steller sea lions were not under thermal stress. Additionally, they used certain areas to preferentially dissipate heat (which parallels findings from [121]). Studies of captive dolphins have shown that the dorsal fin serves as a flexible site for heat dissipation [41,78,102,122], similar to the lesser insulated body surface areas of the Steller sea lions.

In addition to activity level, other factors influencing the use of thermal windows to control thermal balance are body size and water temperature. For example, Hawaiian spinner dolphins (*Stenella longirostris*) rely on activity-induced thermogenesis to maintain their body temperature in waters near their lower critical temperature, which are not uncommon in their habitat range [78,102]. On the other hand, a larger delphinid species, the Pacific bottlenose dolphin, experienced elevated core temperatures after intense exercise, leading to a broader range in body temperatures than the stenothermic Hawaiian spinner dolphins [78]. Despite these differences, the authors concluded that blood flow to the dorsal fin is independent of that to the body trunk for both species. Thus, the interacting effects of exercise and thermoregulation are species- and context-specific based on thermoneutral zones and capacities for thermogenesis.

Unlike studying exercise in a swim flume or large pool, using animals in a captive setting limits our ability to investigate how much the dive response modulates thermal responses because pools are shallow compared to the depths commonly reached by diving marine mammals [27]. Noren et al. [109] took advantage of trained animals in a channel connected to the open ocean and recorded heat flux during dives using hand-held devices. Reduced heat flux at depth (15 m) compared to the surface suggests that heat dissipation is limited until the end of the dive where anticipatory tachycardia occurs. In an exceptional situation, elevated heat flux values at the dorsal fin were observed at depth on a dolphin that had been vigorously active prior to the dive. This indicates there was a momentary override of the dive response to dissipate heat through a thermal window. These findings beg the questions: how often is thermoregulation prioritized during natural diving conditions, and how much volitional control is involved in coordinating these potentially conflicting demands? Translating research with trained dolphins to the natural context can provide a sense of how close they operate to their thermal limits and their physiological capacity to adapt when faced with additional stressors (Figure 2).

### Psychological influences of physiological responses

**Figure 6.**
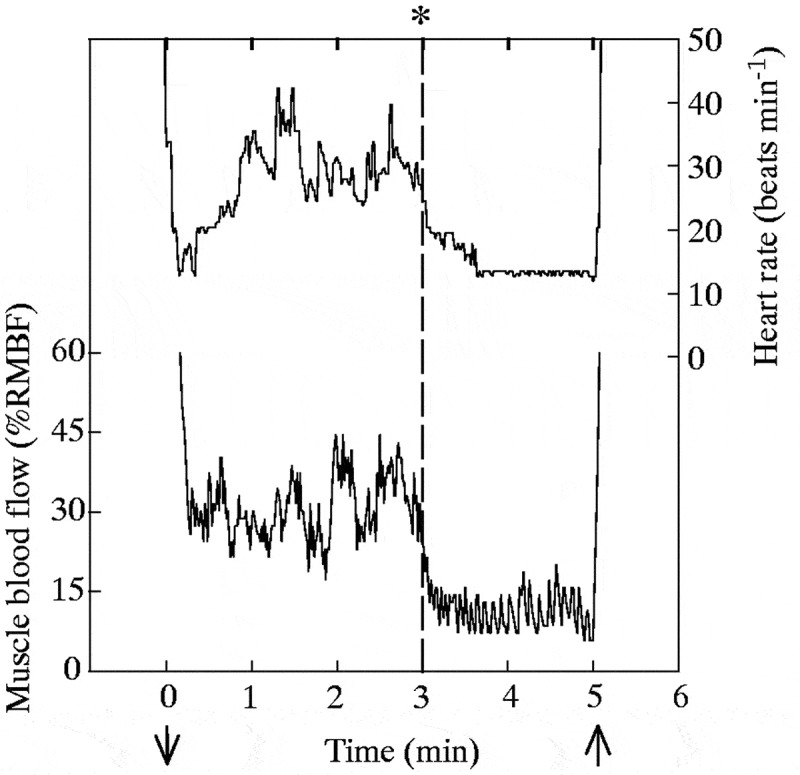
Heart rate and muscle blood flow (as a percentage of mean resting muscle blood flow, %RMBF) of a trained harbor seal (*Phoca vitulina*) during a 5-minute submersion. The seal was accustomed to 3-minute submersions after which its heart rate and muscle blood flow declined to levels similar to naïve seals during submersion. The abrupt change at 3 minutes (dashed line) demonstrates the psychological influences on the physiological responses associated with the dive response. Reprinted with permission from Jobsis et al. 2001. Effects of training on forced submersion responses in harbor seals. The Journal of Experimental Biology, 204: 3877-3885. © The Company of Biologists Ltd.

Researchers have used animals trained or forced to dive or haul out to investigate how anticipatory behavior and psychological influences modulate physiological responses (Figure 2). A diving physiology study compared the dive response in trained versus naïve harbor seals (*Phoca vitulina*) under forced submersions to investigate the effects of anticipation [123]. Compared to naïve seals, trained seals accustomed to 3-minute forced submersions had significantly higher heart rates. They thus maintained greater blood flow to the muscle (measured using laser Doppler flowmetry). In one instance, the submersion was prolonged to 5 minutes without any cues. The heart rate and muscle blood flow profiles show abrupt reductions occurred at precisely 3 minutes into the submersion and were maintained at levels comparable to naïve seals until the end of the submersion (Figure 6). Although forced submersion is far from seals’ natural diving behavior, this and other studies (e.g. [63,124–126]) demonstrated that animals can modify their physiological response when the need arises.

To investigate the degree of control seals exert over heat dissipation through thermal windows, Erdsack et al. [127] used infrared thermography on trained harbor seals. Seals that hauled out voluntarily remained out of the water for several hours and developed thermal windows within minutes regardless of the environmental conditions. Significant amounts of heat dissipation occurred through these thermal windows, which could take several minutes to close once the animal returned to the water resulting in large energy losses. In contrast, when seals hauled out on command during training sessions, they anticipated transitioning between water and land several times. None developed thermal windows, which may be explained by the influence of their psychological state on the autonomic nervous system that regulates peripheral perfusion [128]. By suppressing the formation of thermal windows, amphibious marine mammals minimize excessive heat loss upon returning to water, and thus overall thermoregulatory costs. In addition to demonstrating how physiological demands are balanced to minimize energetic costs, these examples exemplify the value of laboratory studies because sometimes there are not simple parallel approaches in the field.

## Thermal responses during natural diving behavior

Unlike captive studies where animal behavior is manipulated or limited to some extent, wild animals exert control over their activities. During normal, undisturbed diving, it is reasonable to assume that marine mammals that do not frequently haul out are ultimately capable of maintaining thermal balance. Investigating the mechanisms that underlie their physiological thermoregulation requires using remote methods, i.e. biologgers with physiological sensors (Figure 2).

### Temperature regulation as a diving strategy

Studies in diving physiology have used state-of-the-art biologgers to record the impressive breath-hold dives of phocids (true seals) [129–132]. The addition of physiological sensors to time-depth recorders tested the hypothesis that hypothermia while diving enhances aerobic dive capacity and provided insights into body temperature regulation strategies. By measuring arterial and venous blood temperatures as a proxy for core body temperature, Meir and Ponganis [65] demonstrated that a high mean core body temperature (36–37°C) is maintained throughout routine diving behavior (10–30 min) of juvenile northern elephant seals (*Mirounga angustirostris*). Additionally, there was no strong relationship between mean blood temperatures and duration of individual dives. These findings suggest that core hypothermia is not a common strategy in routine dives.

Other studies measuring muscle temperature have reported varying degrees of tissue cooling. For example, there was little to no decline in muscle temperatures in adult Weddell seals despite reductions in aortic temperatures during long dives [130]. On the other hand, a decrease of 4°C occurred in the muscle of a diving immature elephant seal [131]. Both authors concluded that the working muscle was either hypometabolic or maintained some level of perfusion to allow dissipation of locally generated heat. They also noted that peripheral cooling extends beyond the insulation into muscle [9,131]. The degree to which this occurs and where core body temperature measurements are taken influence how the data are interpreted to align with different thermal strategies [66,133]. As emphasized in these studies, the heterogeneity of body temperature precludes obtaining a representative whole-body temperature from just one location [133]. Multiple measurements at different locations enable a more holistic understanding of heat distribution throughout the body and the mechanisms contributing to heat transfer.

### Peripheral perfusion patterns: Insights from modeling

Even if multiple simultaneous measurements are possible, numerous factors are impossible to control in the wild, making it difficult to decipher which variables are independent or dependent. To develop a mechanistic understanding of thermal dynamics in a swimming and diving animal, modeling approaches can simulate background variation while testing specific hypotheses regarding variables or parameters of interest. With a time-series of *in situ* measurements to compare model outputs, Boyd [33] modeled peripheral circulation as “on-off” states in a heat balance model to gain insight into the processes affecting the temporal dynamics of the temperature gradient between the water and skin of diving Antarctic fur seals (*Arctocephalus gazella*). During dives, the temperature gradient decreased and then increased toward the end of the dive. Transient increases occurred at the bottom of the dive and were attributed to changes in activity. Their model tracked this variability in the temperature gradient reasonably well. Discrepancies between the model output and *in situ* measurements were likely due to an overly simplistic representation of the regulation of perfusion as an abrupt switch between two states rather than a more graded process.

### Fine-scale changes in peripheral heat flow while diving

Unlike fur seals which maintain a large temperature gradient between their skin and water [33], marine mammals relying on blubber will experience more profound variations in their insulation due to changes in peripheral perfusion in response to water temperature and as a result of the dive response. Laboratory experiments have demonstrated the former by measuring physiological variables while exposing marine mammals to temperature-controlled water baths [9,34,78,106]. To explore the latter, we recorded heat flux and peripheral temperatures in freely diving juvenile northern elephant seals using custom-made biologgers.

**Figure 7.**
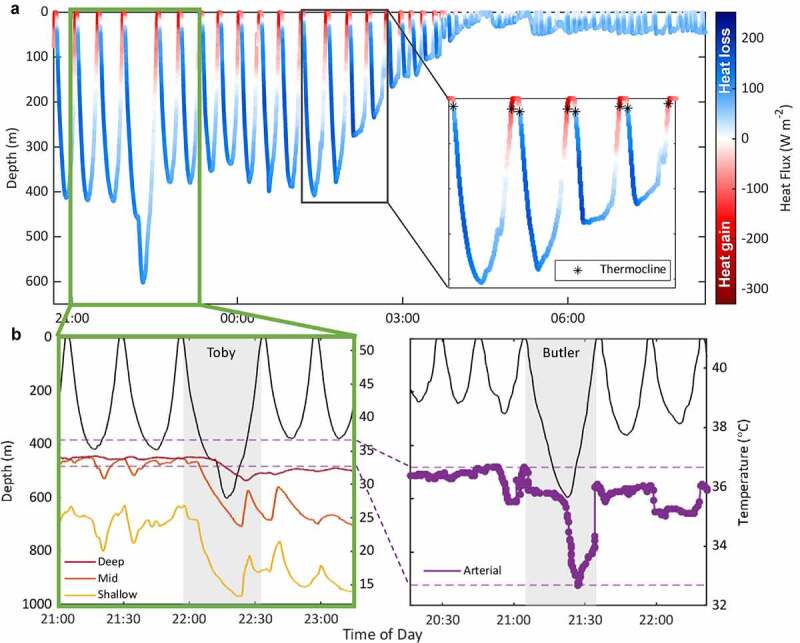
The dive profile of Toby, a translocated juvenile northern elephant seal (*Mirounga angustirostris*), with the color indicating the direction of heat flux (red = heat gain, blue = heat loss) throughout the dive. The inset shows four deep dives (>200 m) and how the transition from heat gain to heat loss occurs near the thermocline (denoted with asterisks) on the descent, but the opposite transition occurs at deeper depths during the ascent. Heat flux values [W m^−2^] in (a) are preliminary values pending post-deployment calibrations. Raw voltage output has been corrected for the added thermal resistance of the sensor and attachment mechanism (determined experimentally) as well as the unique sensor’s calibration constant (provided by the manufacturer). A section of the dive profile denoted by the green box containing the seal’s deepest dive in (a) is shown in greater detail (b, left) with temperature profiles at three depths within the blubber layer (deep = red, mid = orange, shallow = yellow). The drop in blubber temperature during the deep dive (b, left) is analogous to the drop in arterial temperature (b, right) recorded in Butler, a translocated juvenile northern elephant seal by [65], although arterial temperature declined more abruptly. Both blubber and arterial temperatures rewarmed before the end of the dive, and arterial temperatures remained comparatively warmer (note the difference in temperature range of the y-axes; the range of arterial temperatures is shown on both figures with the purple dashed lines). The figure showing Butler’s data is modified from Meir and Ponganis, 2010. Blood Temperature Profiles of Diving Elephant Seals. Physiological and Biochemical Zoology, 83(3): 531–540. © The University of Chicago Press.

Heat flux showed consistent within-dive patterns: seals generally lost heat throughout most of the dive and gained heat throughout the short post-dive intervals (Figure 7a). A departure from this pattern occurred during shallow diving bouts. Instead of gaining heat at the surface, the seals lost heat throughout the ascent and surface interval (Figure 7a). This thermal response during shallow diving bouts may occur because their shallow dives are in relatively warm water (>10°C). As a result, they experience smaller changes in water temperature within a dive cycle. Higher arterial temperatures during short, shallow diving bouts observed by Meir and Ponganis [65] also suggest a greater need to dissipate heat than conserve heat while in relatively warm waters to maintain thermal homeostasis. When they dive deeper into cold waters (~5°C), their skin temperature drops to within a few degrees of water temperature. During the latter portion of their ascent, their skin is colder than the surrounding water, which reverses the temperature gradient resulting in a transition to gaining heat. How these different heat flux patterns ultimately affect their thermal balance – and the relative importance of activity and body size on thermal dynamics – is currently being investigated.

Another variation in pattern is the transition from heat gain to heat loss occurring near the thermocline on the descent, while the opposite transition occurred at deeper and more variable depths on the ascent (Figure 7a). Whether dive conditions and an individual’s dive capacity contribute to the earlier transition remains to be tested but aligns with temperature profiles on deep dives suggesting increased blood flow and perfusion of the periphery during the ascent (Figure 7b). Arterial blood temperature [65] and blubber temperatures on long dives (≥30 mins) declined but always rewarmed before the end of the dive. This is consistent with the anticipatory tachycardia and the resulting redistribution of blood flow that marine mammals experience during the dive ascent [112,131,134,135].

**Figure 8.**
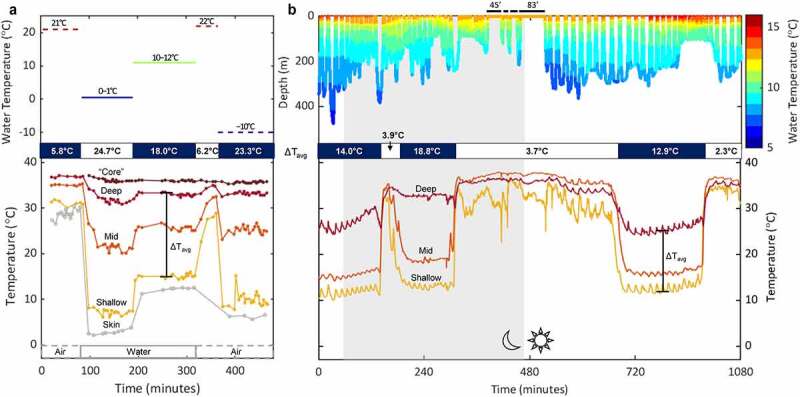
Skin and tissue temperature measurements of a harbor seal (*Phoca vitulina*) in air and water at temperatures indicated by the dashed and solid line segments, respectively (a). Tissue temperatures were measured at 3 mm (shallow), 13 mm (mid), 23 mm (deep), and 42 mm (“core”), which included the blubber layer (<25 mm thick) and deeper tissue. The seal was allowed to equilibrate for at least one hour at each ambient temperature while recording measurements every 4 minutes. Adapted from Hart and Irving, 1959. The energetics of harbor seals in air and water with special considerations of seasonal changes. Canadian Journal of Zoology, 37: 447–457. © Canadian Science Publishing. The dive profile of a translocated juvenile northern elephant seal (*Mirounga angustirostris*) that dove continuously for 7 days (18 hours shown in figure) and experienced water temperatures between 5–16°C (b, top). Four consecutive extended surface intervals are marked with thick black bars along with the duration (in minutes) of the first and last extended surface intervals. Temperature profiles at three depths within the blubber layer (deep = red, mid = orange, shallow = yellow) reveal how the temperature gradient within the blubber layer (ΔT_avg_ = average(T_deep_−T_shallow_)) undergoes large variations across dives (b, bottom). The cyclical nature of diving also results in smaller fluctuations within dives that is not observed in the resting harbor seal in (a) .

Blubber temperatures showed a distinct gradient, with greater variation across dives than within dives (Figure 8b). While the minimum temperature varied among individuals, deep blubber temperatures fell as low as 22.9°C during deep dives (>200 m), resulting in a blubber gradient (ΔT_avg_) of 14.2°C. During shallow diving (<100 m) and extended surface intervals, the blubber gradient dropped to <5°C. The transition from a large blubber gradient at low temperatures to a smaller gradient at higher temperatures occurred more abruptly than the opposite transition (Figure 8b). Simultaneous changes in diving behavior were not apparent in either case. This suggests that these thermal responses were not simply due to passive warming or cooling from changing water temperatures but were actively regulated through vasomotor changes.

Similarly, Hart and Irving [9] attributed the rapid changes observed in subcutaneous temperatures of captive harbor seals in response to changing ambient temperatures to active regulation (Figure 8a). It is worth comparing blubber temperature data from captive harbor seals (Figure 8a) to wild northern elephant seals (Figure 8b) to highlight how similar measurements taken 50 years apart yielded valuable insights into peripheral perfusion as a fundamental physiological mechanism in marine mammal thermoregulation. When animals are exposed to constant air or water temperature within their thermal limits, their thermal responses will stabilize after some time. On the other hand, the cyclical nature of diving results in more complex changes mainly in the peripheral body, which will consequently affect heat transfer. While minimum water temperature (at depths >100 m) differed by less than 5°C across dives, shallow blubber temperatures varied as much as 26°C. By measuring similar physiological variables on a freely diving seal, our study replicates past controlled experiments in an uncontrolled setting, incorporating an essential behavioral component (i.e. diving). Our field research builds on previous laboratory findings and extends our understanding of their thermal responses to the natural context.

Our data from freely diving elephant seals further demonstrates the utility of combining multiple continuous measurements at fine resolution to provide insight into the complex physiological responses to the interacting demands of diving and thermoregulation in marine mammals. Together, the heat flux patterns and temperature profiles indicate that allowing peripheral cooling prevents conflict with the dive response, especially during deep-diving bouts. During the short surface intervals (2–3 mins), reperfusion of the periphery associated with a relaxation of the dive response increases blubber temperatures slightly (mean increase of <1°C at all blubber depths, max of 5°C in the shallow blubber). Still, longer periods of shallow diving or extended surface intervals seem to be required to return the deep blubber close to normothermia (35.3 ± 2.1°C). Whether these cyclical changes in perfusion contribute to maintaining whole-body thermal balance or increase thermoregulatory costs is still unknown. Nonetheless, our data supports the idea that regional heterothermy – more specifically, peripheral hypothermia – is preferred over strict homeothermy as a thermal strategy and may offer a compromise between thermal and oxygen demands for continuously diving marine mammals, like elephant seals.

## Considerations for future progress in the field

Understanding how marine mammals survive in such a thermally challenging environment is a complex subject that requires a multi-pronged approach, particularly given the difficulty of studying marine mammals. Here we focused on laboratory or field studies that have provided insight into the role of peripheral perfusion as a physiological mechanism that directly regulates or indirectly influences heat distribution within and across the body. We acknowledge that this is most relevant for marine mammals that primarily rely on blubber for insulation. Integrating *in vivo* and field approaches will deepen our understanding of how thermal responses interact with conflicting physiological demands, such as the dive response. Used together, these approaches will provide ecologically relevant insight into an important but understudied aspect of marine mammal physiology and energetics (Figure 2).

### Outstanding gaps: The many roles of peripheral perfusion

Future work should address other conflicting physiological demands that require peripheral perfusion and their interactions with thermoregulation. For instance, molting is a necessary phenomenon that requires perfusion of the skin to replace old skin and fur. The form and phenology of molting vary widely, from annual catastrophic molting to continuous gradual molting. It often depends on whether animals can find thermal refugia to minimize the energetic costs of increased heat loss associated with skin perfusion [136–139]. Similarly, wound healing requires perfusion of the injured site, potentially leading to a tradeoff in short-term energetic costs and long-term health and survival. Haul-out periods for amphibious species, or seasonal residency at warmer latitudes for long-distance migrators, allow for temporal separation of these physiological demands that would otherwise increase thermoregulatory costs. Finally, unlike fur, blubber is a living tissue that serves as an energy store, and depositing or metabolizing lipid stores also requires perfusion of this layer [32,140,141]. Thus, in addition to the dual role associated with thermoregulation and diving that was the primary focus of this review, peripheral perfusion is critical for many other physiological processes. How these conflicting demands interact with each other warrants further investigation.

### Challenging but insightful measurements

While temperature measurements are commonly used in biologging studies and have various applications (e.g. stomach temperature telemetry in foraging ecology studies [142,143]), heat flux has generally been limited to studies directly interested in thermal physiology. Nevertheless, heat flux data have proven to be quite informative. Still, the nature of the measurements is more complex than temperature measurements. The sensor provides thermal resistance at the measurement site, resulting in heat flux values that do not accurately represent heat flux from adjacent skin surfaces [144]. Therefore, it is critical to determine a correction factor for the sensor and the attachment mechanism [38,41,145–148]. Additionally, observations of high variability in heat flux values both among and within individuals are common, which should caution the quantitative interpretation of heat flux values [41,149].

Some studies have also noted large changes in heat flux associated with small temperature changes between the skin and water. Whether this discrepancy has a physiological underpinning warrants further investigation [109,110,145]. Changes in convective heat transfer via blood flow may explain these periodic increases in heat flow at localized skin surface areas [15,56,150]. However, obtaining a direct measure of peripheral blood flow is difficult. Blood flow measurements have only been done on a few captive animals [123,124,151–156]. While many direct and indirect methods of measuring blood flow exist and improve human applications (see [47,157] for methodological reviews), their sensitivity to motion is a significant barrier to their use on free-ranging animals.

As demonstrated in freely diving juvenile elephant seals, blubber temperature is more readily measured than blood flow and is thus a good proxy for peripheral perfusion. Using indirect measures, such as peripheral temperature and derivatives, to infer peripheral perfusion is not a novel approach (examples primarily from the seabird literature, e.g. [140,141,158–160]) and even has diagnostic potential in biomedical applications [161]. Unlike the skin surface, blubber is not directly affected by external influences, such as convective heat loss from water flow. Instead, blubber temperatures reflect internal processes that serve to maintain homeostasis.

### Integrating behavioral and physiological thermoregulation

While this review focused on the critical role of peripheral perfusion, it is essential to underscore that behavior is generally the first line of defense for maintaining thermal homeostasis. Schlader [162] noted less than a decade ago that behavioral thermoregulation is often neglected in human studies, even more so than in animal studies. Animals outside their thermoneutral zone employ more energetically costly strategies such as sweating or shivering to restore thermal balance (Figure 4). However, conflicting physiological demands may prevent marine mammals from employing these mechanisms. For example, the need to conserve water may explain why California sea lions (*Zalophus californianus*) neither pant nor sweat despite having functional sweat glands, unlike elephant seals. Rather, both species seem to rely on behavior to deal with the warm air temperatures within their home range and prevent heat stress [163,164]. Similarly, diving marine mammals cannot employ sweating or shivering while diving [101]. Therefore, they must either avoid exceeding their upper and lower critical temperatures or utilize behavioral strategies that increase their sensible heat exchange effectiveness (Figure 4).

Natural conditions may allow behavioral interventions to avoid reaching the boundaries of the thermoneutral zone and remain in their thermal comfort zone [162,165] (Figure 4). Within this zone, marine mammals combine physiological mechanisms and behavioral strategies on different timescales to maintain thermal balance. For example, sea otters minimize thermoregulatory costs by spending a significant amount of time floating at the surface and balancing thermal substitution from activity and the heat increment of feeding [166]. Marine mammals that haul out can switch between the two media as a form of behavioral thermoregulation [23,26,57,164]. Deeper diving marine mammals may modify their diving behavior to take advantage of the steep gradients in water temperature to adjust their thermal balance (as observed in ectothermic and/or regional endothermic vertebrates, pelagic tuna and sharks [167–171]). While regulating peripheral perfusion provides a faster mechanism for adjusting heat transfer, behavioral strategies, such as extended surface intervals, may allow for slower but larger adjustments in overall heat balance that would otherwise conflict with the dive response, especially during non-routine behavior.

To truly integrate our understanding of behavioral and physiological thermoregulation, we need to study wild animals minimally disturbed and behaving naturally (Figure 2). Most behavioral thermoregulatory studies of wild marine mammals were carried out on animals on the beach [25,57,164,172–174] or at the water’s surface near the coastline [174] (Figure 1). With the rapid integration of inertial measurement units into biologgers, sophisticated interpretations of their non-observable behavior at-sea are now possible [175,176]. These can be combined with measurements relevant to thermal physiology to determine how activity level and activity-induced thermogenesis influence peripheral dynamics and heat balance.

Studies of how behavior and intense activity modify thermal balance have implications for understanding the thermoregulatory costs of marine mammals exposed to anthropogenic disturbances. Disturbances, such as fisheries interactions, ship traffic, or sonar, can lead to altered diving behavior or haul-out patterns (Figure 2). For example, simulated sonar has been shown to cause marine mammals to prolong their dives [177]. If the dive response is modulated by dive conditions, these unanticipated prolongations will reduce heart rate and peripheral perfusion. Such cardiovascular adjustments would result in secondary consequences for heat dissipation or potentially a complete override of thermoregulatory demands.

To investigate these issues related to conservation, we need to study their thermoregulation in an ecophysiologically relevant context (Figure 2). By performing controlled exposure experiments on wild animals [178–180], we can quantify biologically significant and context-dependent responses to disturbances (e.g. [181]). Minimizing or avoiding activities known to increase the susceptibility of marine mammals to thermal imbalance will help reduce the cumulative impacts of anthropogenic stressors on marine mammals. However, we must first establish a baseline understanding of their thermal physiology during natural behavior before understanding the pathophysiology associated with disturbed behavior.

## Supplementary Material

Supplemental Material

## Data Availability

The data used to create Figures 7 and Figure 8 are openly available in Dryad at https://doi.org/10.7291/D1M09M where additional information regarding methods for data collection is also present.

## Note

1. The Krogh Principle states that “for a large number of problems there will be some animal of choice or a few such animals on which it can be most conveniently studied” [182]. We consider marine mammals to be “Krogh organisms” for studying thermal physiology because they have noteworthy, or even extreme, thermal adaptations (e.g. morphological traits and physiological mechanisms) [183].
