# Supplementary material for: Advances in thermal physiology of diving marine mammals: The dual role of peripheral perfusion
Source: Temperature (Austin). 2021 Dec 18;9(1):46–66. doi: 10.1080/23328940.2021.1988817 (PMC9154795; doi:10.1080/23328940.2021.1988817)
Supplement: Supplemental Material [file KTMP_A_1988817_SM5650.pdf]

Supplementary File

Publications organized by year since 1940 relevant to the thermal physiology of marine mammals. Refer to the caption of Figure 1 for an explanation of what types of publications were included, how they were classified, and how the different categories are defined. The following are indicated in the 'Notes' column: (1) studies that are relevant but traditionally considered a study on diving physiology; (2) whether field studies were performed on land or in the water; (3) the type of biotelemetry technology used for studies that used biotelemetry to study physiology and/or behavior in the lab or field. Biotelemetry methods for (1) physiology: STP = stomach temperature pill (a temperature-sensitive radio transmitter that is ingested), IRT = infrared thermography (used for skin temperature unless otherwise stated), ECG = electrocardiogram (heart rate), HF = heat flux sensor, TR = thermistor (temperpature location specified), TC = thermocouple (temperature location specified); (2) behavior: TDR = time-depth recorder (diving behavior), SAT = satellite tag (location/movement data), GPS = global positioning satellite tag (location/movement data), VHF = very high frequency transmitter (location/movement data), CTD = conductivity-temperature-depth tag (*in situ* water temperature).

**Publication**					**Study Setting**			**Animal State**							**Biotelemetry**		**Notes**
	Publication	Title		Year	Field	Lab	Modeling	Free-ranging	Trained	Captive	Restrained	Peri-mortem	Carcass	*Ex vivo*	Physiology	Behavior	
**1940-1949**																	
1	Scholander, PF; Irving, Laurence; Grinnell, SW	On the Temperature and Metabolism of the seal during diving	Journal of Cellular and Comparative Physiology	1942		X					X						diving physiology study
2	Parry, DA;	The structure of whale blubber, and a discussion of its thermal properties	Journal of Cell Science	1949		X								X			
**1950-1959**																	
3	Scholander, PF; Schevill, WE	Counter-Current Vascular Heat Exchange in the Fins of Whales	Journal of Applied Physiology	1955		X								X			
4	Bartholomew, GA; Wilke, F	Body temperature in the northern fur seal, *Callorhinus ursinus*	Journal of Mammalogy	1956	X						X	X					on land
5	Irving, L; Hart, JS	The metabolism and insulation of seals as bare-skinned mammals in cold water	Canadian Journal of Zoology	1957		X					X						
6	Hart, JS; Irving, L	The energetics of harbor seals in air and in water with special consideration of seasonal changes	Canadian Journal of Zoology	1959		X					X						
**1960-1969**																	
7	Irving, L; Peyton, LJ; Bahn, CH; Peterson, RS	Regulation of temperature in fur seals	Physiological Zoology	1962	X	X					X	X					
8	Bryden, MM	Insulating capacity of the subcutaneous fat of the southern elephant seal	Nature	1964		X								X			
9	Ling, JK	Functional significance of sweat glands and sebaceous glands in seals	Nature	1965		X								X			
10	Ling, JK	The skin and hair of the Southern Elephant Seal *Mirounga leonina* (L.) III. morphology of the adult integument	Australian Journal of Zoology	1968		X								X			
**1970-1979**																	
11	Tarasoff, FJ; Fisher, HD	Anatomy of the hind flippers of two species of seals with reference to thermoregulation	Canadian Journal of Zoology	1970		X								X			
12	Hampton, IFG; Whittow, GC; Szekerczes, J; Rutherford, S	Heat transfer and body temperature in the Atlantic bottlenosed dolphin, *Tursiops truncatus*	The International Journal of Biometerology	1971		X			X						X		STP
13	McGinnis, SM; Southworth, TP	Thermoregulation in the northern elephant seal, *Mirounga angustirostris*	Comparative Biochemistry and Physiology	1971	X	X		X		X					X		STP, implanted radio telemeters ($T_{subcutaneous}$), field component on land
14	White, FN; Odell, DK	Thermoregulatory behavior of the northern elephant seal, *Mirounga angustirostris*	Journal of Mammalogy	1971	X			X							X		STP, on land

Publication					Study Setting			Animal State							Biotelemetry		Notes
	Publication	Title		Year	Field	Lab	Modeling	Free-ranging	Trained	Captive	Restrained	Peri-mortem	Carcass	*Ex vivo*	Physiology	Behavior	
15	McGinnis, SM; Whittow, GC; Ohata, CA; Huber, H	Body heat dissipation and conservation in two species of dolphins	Comparative Biochemistry and Physiology Part A: Physiology	1972		X				X					X		STP, implanted radio telemeters ($T_{subcutaneous}$)
16	Ohata, CA; Matsuura, DT; Whittow, GC; Tinker, SW	Diurnal Rhythm of Body Temperature in the Hawaiian Monk Seal (*Monachus schauinslandi*)	Pacific Science	1972		X				X					X		STP
17	Whittow, GC; Matsuura, DT; Lin, YC	Temperature regulation in the California sea lion (*Zalophus californianus*)	Physiological Zoology	1972		X								X			
18	Gentry, RL	Thermoregulatory behavior of eared seals	Behaviour	1973	X			X									on land
19	Matsuura, DT; Whittow, GC	Evaporative heat loss in the California sea lion and harbor seal	Comparative Biochemistry and Physiology Part A: Physiology	1974		X				X	X				X		rectal telemetry capsule
20	Morrison, P; Rosenmann, M; Estes, JA	Metabolism and thermoregulation in the sea otter	Physiological Zoology	1974		X				X	X						
21	Odell, DK	Behavioral thermoregulation in the California sea lion	Behavioral Biology	1974	X			X									on land
22	Ohata, CA; Whittow, GC	Conductive heat loss to sand in California sea lions and a harbor seal	Comparative Biochemistry and Physiology	1974		X				X							
23	Whittow, GC; Hampton, IFG; Matsuura, DT; Ohata, CA; Smith, RM; Allen, JF	Body Temperature of Three Species of Whales	Journal of Mammalogy	1974		X				X					X		STP, IRT
24	Luecke, RH; Natarajan, V; South, RE	A mathematical biothermal model of the California sea lion	Journal of Thermal Biology	1975			X										
25	McGinnis, SM	Peripheral Heat Exchange in Phocids	Rapp. P.-v. Reun. Cons. int. Explor. Mer.	1975	X	X		X		X					X		STP, field component performed on land
26	Miller, K; Irving, L	Metabolism and temperature regulation in young harbor seals *Phoca vitulina richardi*	American Journal of Physiology-Legacy Content	1975		X				X							
27	Molyneux, GS; Bryden, MM	Arteriovenous anastomoses in the skin of the Weddell seal, *Leptonychotes weddelli*	Science	1975		X								X			
28	Whittow, GC; Szekerczes, J; Kridler, E; Olsen, DL	Skin structure of the Hawaiian monk seal (*Monachus schauinslandi*)	Pacific Science	1975		X								X			
29	Hampton, IFG; Whittow, GC	Body temperature and heat exchange in the Hawaiian spinner dolphin, *Stenella longirostris*	Comparative Biochemistry and Physiology Part A: Physiology	1976		X			X	X					X		IRT
30	Miller, K; Rosenmann, M; Morrison, P	Oxygen uptake and temperature regulation of young harbor seals (*Phoca vitulina richardi*) in water	Comparative Biochemistry and Physiology Part A: Physiology	1976		X				X							

Publication					Study Setting			Animal State							Biotelemetry		Notes
	Publication	Title		Year	Field	Lab	Modeling	Free-ranging	Trained	Captive	Restrained	Peri-mortem	Carcass	*Ex vivo*	Physiology	Behavior	
31	Hammel, HT; Elsner, RW; Heller, HC; Maggert, JA; Bainton, CR	Thermoregulatory responses to altering hypothalamic temperature in the harbor seal	American Journal of Physiology. Regulatory, Integrative and Comparative Physiology	1977		X					X						
32	Ohata, CA; Miller, LK	Northern fur seal thermoregulation: Thermal responses to forced activity on land	Journal of Thermal Biology	1977	X							X					on land
33	Ohata, CA; Miller, LK	Some temperature responses of northern fur seal (Callorhinus ursinus) pups	Journal of Mammalogy	1977	X						X						on land
34	Ohata, CA; Miller, LK; Kajimura, H	Northern fur seal thermoregulation: Thermal responses to pelagic conditions	Journal of Thermal Biology	1977	X							X					in water
35	Bryden, MM; Molyneux, GS	Arteriovenous anastomoses in the skin of seals. II. The California sea lion *Zalophus californianus* and the northern fur seal *Callorhinus ursinus* (Pinnipedia: Otariidae)	The Anatomical Record	1978		X								X			
36	Molyneux, GS; Bryden, MM	Arteriovenous anastomoses in the skin of seals. I. The Weddell Seal *Leptonychotes weddelli* and the Elephant Seal *Mirounga leonina* (Pinnipedia: Phocidae)	The Anatomical Record	1978		X								X			
37	Øritsland, NA; Ronald, K	Aspects of temperature regulation in harp seal pups evaluated by in vivo experiments and computer simulations	Acta Physiologica Scandinavica	1978		X	X				X						
38	Blix, AS; Grav, H; Ronald, K	Some aspects of temperature regulation in newborn harp seal pups	American Journal of Physiology-Regulatory, Integrative and Comparative Physiology	1979		X								X			
39	Blix, AS; Miller, LK; Keyes, MC; Grav, HJ; Elsner, R	Newborn northern fur seals (*Callorhinus ursinus*)--do they suffer from cold?	American Journal of Physiology-Regulatory, Integrative and Comparative Physiology	1979		X				X	X		X	X			
40	Gallivan, GJ; Ronald, K	Temperature regulation in freely diving harp seals (*Phoca groenlandica*)	Canadian journal of Zoology	1979		X				X					X		STP, diving physiology study
41	Zapol, WM; Liggins, GC; Schneider, RC; Qvist, J; Snider, MT; Creasy, RK; Hochachka, PW	Regional blood flow during simulated diving in the conscious Weddell seal	Journal of Applied Physiology	1979		X					X						diving physiology study
**1980-1989**																	
42	Kooyman, GL; Wahrenbrock, EA; Castellini, MA; Davis, RW; Sinnett, EE	Aerobic and anaerobic metabolism during voluntary diving in Weddell seals: Evidence of preferred pathways from blood chemsitry and behavior	Journal of Comparative Physiology B	1980	X			X			X					X	TDR, diving physiology study, in water
43	Costa, DP; Kooyman, GL	Oxygen consumption, thermoregulation, and the effect of fur oiling and washing on the sea otter, *Enhydra lutris*	Canadian Journal of Zoology	1982		X				X				X	X		STP, implanted radio telemeters ($T_{subcutaneous}$)

Publication					Study Setting			Animal State							Biotelemetry		Notes
	Publication	Title		Year	Field	Lab	Modeling	Free-ranging	Trained	Captive	Restrained	Peri-mortem	Carcass	*Ex vivo*	Physiology	Behavior	
44	Blix, AA; Fay, FH; Ronald, K	On testicular cooling in phocid seals	Polar Research	1983	X	X						X	X				field component on land
45	Gallivan, GJ; Best, RC; Kanwisher, JW	Temperature regulation in the Amazonian manatee *Trichechus inunguis*	Physiological Zoology	1983		X				X					X		STP
46	Irvine, AB	Manatee metabolism and its influence on distribution in Florida	Biological Conservation	1983		X				X					X		STP
47	Costa, DP; Kooyman, GL	Contribution of specific dynamic action to heat balance and thermoregulation in the sea otter *Enhydra lutris*	Physiological Zoology	1984		X				X							
48	Brodie, P; Paasche, A	Thermoregulation and energetics of fin and sei whales based on postmortem, stratified temperature measurements	Canadian Journal of Zoology	1985	X							X					in water
49	Limberger, D; Trillmich, F; Biebach, H; Stevenson, RD	Temperature regulation and microhabitat choice by free-ranging Galapagos fur seal pups (*Arctocephalus galapagoensis*)	Oecologia	1986	X			X							X		STP, IRT, on land
50	Yasui, WY; Gaskin, DE	Energy budget of a small cetacean, the harbour porpoise, *Phocoena phocoena* (L.)	Ophelia	1986		X	X							X			
51	Folkow, LP; Blix, AS	Nasal heat and water exchange in gray seals	American Journal of Physiology-Regulatory, Integrative and Comparative Physiology	1987		X			X								
52	Hill, RD; Schneider, RC; Liggins, GC; Schuette, AH; Elliott, RL; Guppy, M; Hochachka, PW; Qvist, J; Falke, KJ; Zapol, WM; Lig-Gins, GC	Heart rate and body temperature during free diving of Weddell seals	The American Physiological Society	1987	X			X							X	X	ECG, blood samples, thermistor-tipped catheter (T_{blood}), TDR, VHF, diving physiology study, in water
53	Thompson, SD; Ono, KA; Oftedal, OT; Boness, DJ	Thermoregulation and resting metabolic rate of California sea lion (*Zalophus californianus*) pups	Physiological Zoology	1987	X						X						on land
54	Campagna, C; Le Boeuf, BJ	Thermoregulatory behaviour of southern sea lions and its effect on mating strategies	Behaviour	1988	X			X									on land
55	Ryg, M; Smith, TG; Øritsland, NA	Thermal significance of the topographical distribution of blubber in ringed seals (*Phoca hispida*)	Canadian Journal of Fisheries and Aquatic Sciences	1988		X						X	X	X			
56	Folkow, LP; Blix, AS	Thermoregulatory control of expired air temperature in diving harp seals	American Journal of Physiology-Regulatory, Integrative and	1989		X			X		X						diving physiology study
57	Kasting, NW; Adderley, SAL; Safford, T; Hewlett, KG	Thermoregulation in beluga (*Delphinapterus leucas*) and killer (*Orcinus orca*) whales	Physiological Zoology	1989		X			X								
**1990-1999**																	
58	Doidge, DW	Integumentary heat loss and blubber distribution in the beluga, *Delphinapterus leucas* , with comparisons to the narwhal, *Monodon monoceros*	Canadian Bulletin of Fisheries and Aquatic Science	1990		X							X	X			

Publication					Study Setting			Animal State							Biotelemetry		Notes
	Publication	Title		Year	Field	Lab	Modeling	Free-ranging	Trained	Captive	Restrained	Peri-mortem	Carcass	*Ex vivo*	Physiology	Behavior	
59	Hokkanen, JEI	Temperature regulation of marine mammals	Journal of Theoretical Biology	1990			X										
60	Innes, S; Worthy, GAJ.; Lavigne, DM; Ronald, K	Surface area of phocid seals	Canadian Journal of Zoology	1990		X								X			
61	Lavigne, DM; Innes, S; Worthy, GAJ; Edwards, EF	Lower critical temperatures of blue whales, *Balaenoptera musculus*	Journal of Theoretical Biology	1990			X										
62	Øristland, NA; Markussen, NH	Outline of a physiologically based model for population energetics	Ecological Modelling	1990			X										
63	Ryg, M; Lydersen, C; Markussen, NH; Smith, TG; Oritsland, NA	Estimating the blubber content of phocid seals	Canadian Journal of Fisheries and Aquatic Sciences	1990		X							X	X			
64	Trites, AW	Thermal budgets and climate spaces: the impact of weather on the survival of Galapagos (*Arctocephalus galapagoensis Heller*) and northern fur seal pups (*Callorhinus ursinus L.*)	Functional Ecology	1990			X										
65	Worthy, GAJ; Edwards, EF	Morphometric and biochemical factors affecting heat loss in a small temperate cetacean (*Phocoena phocoena*) and a small tropical cetacean (*Stenella attenuata*)	Physiological Zoology	1990		X							X	X			
66	Francis, JM; Boness, DJ	The effect of thermoregulatory behaviour on the mating system of the Juan Fernandez fur seal, *Arctocephalus philippii*	Behaviour	1991	X			X									on land
67	Little, GJ	Thyroid morphology and function and its role in thermoregulation in the newborn southern elephant seal (*Mirounga leonina*) at Macquarie Island	Journal of Anatomy	1991	X	X					X			X			field component on land
68	Smith, TG; Hammill, MO; Taugbøl, G	A review of the developmental, behavioural and physiological adaptations of the ringed seal, *Phoca hispida* , to life in the Arctic winter	Arctic	1991		X					X		X	X			
69	Worthy, GAJ	Insulation and thermal balance of fasting harp and grey seal pups	Comparative Biochemistry and Physiology Part A: Physiology	1991		X				X		X		X	X		STP
70	Cuyler, LC; Wiulsrød, R; Øritsland, NA	Thermal infrared radiation from free living whales	Marine Mammal Science	1992	X			X							X		IRT, in water
71	Folkow, LP; Blix, AS	Metabolic rates of minke whales (*Balaenoptera acutorostrata*) in cold water	Acta Physiologica Scandinavica	1992	X	X		X				X					field component in water
72	Rommel, SA; Pabst, DA; McLellan, WA; Mead, JG; Potter, CW	Anatomical evidence for a countercurrent heat exchanger associated with dolphin testes	The Anatomical Record	1992		X							X	X			
73	Ponganis, PJ; Kooyman, GL; Castellini, MA; Ponganis, EP; Ponganis, KV	Muscle temperature and swim velocity profiles during diving in a Weddell seal, *Leptonychotes weddellii*	Journal of Experimental Biology	1993	X			X							X	X	percutaneous TR_{muscle}, TDR, swim velocity, diving physiology study, in water

Publication					Study Setting			Animal State							Biotelemetry		Notes
	Publication	Title		Year	Field	Lab	Modeling	Free-ranging	Trained	Captive	Restrained	Peri-mortem	Carcass	*Ex vivo*	Physiology	Behavior	
74	Rommel, SA; Pabst, DA; McLellan, WA	Functional morphology of the vascular plexuses associated with the cetacean uterus	The Anatomical Record	1993		X							X	X			
75	Ryg, M; Lydersen, C; Knutsen, LØ; Bjørge, A; Smith, TG; Øritsland, NA	Scaling of insulation in seals and whales	Journal of Zoology	1993		X							X	X			
76	Watts, P; Hansen, S; Lavigne, DM	Models of heat loss by marine mammals: thermoregulation below the zone of irrelevance	Journal of Theoretical Biology	1993			X										
77	Kvadsheim, PH; Folkow, LP; Blix, AS	A new device for measurement of the thermal conductivity of fur and blubber	Journal of Thermal Biology	1994		X								X			
78	Rommel, SA; Pabst, DA; Mclellan, WA; Williams, TM; Friedl, WA	Temperature regulation of the testes of the bottlenose dolphin (*Tursiops truncatus*): evidence from colonic temperatures	Journal of Comparative Physiology B	1994		X			X								
79	Beck, GG; Smith, TG	Distribution of blubber in the northwest Atlantic harp seal, *Phoca groenlandica*	Canadian Journal of Zoology	1995		X							X	X	X		
80	Boily, P	Theoretical heat flux in water and habitat selection of phocid seals and beluga whales during the annual molt	Journal of Theoretical Biology	1995			X										
81	Hansen, S; Lavigne, DM; Innes, S	Energy metabolism and thermoregulation in juvenile harbor seals (*Phoca vitulina*) in air	Physiological Zoology	1995		X				X					X		STP
82	Kastelein, RA; Van der Sijs, SJ; Staal, C; Nieuwstraten, SH	Blubber thickness in harbour porpoises (*Phocoena phocoena*)	Food Consumption and Growth of Marine Mammals	1995		X				X					X		IRT
83	Little, GJ	Body temperature in the newborn southern elephant seal, *Mirounga leonina* , at Macquarie Island	Marine Mammal Science	1995	X						X						on land
84	Pabst, DA; Rommel, SA; Mclellan, WA; Williams, TM; Rowles, TK	Thermoregulation of the intra-abdominal testes of the bottlenose dolphin (*Tursiops truncatus*) during exercise	The Journal of Experimental Biology	1995		X			X						X		TC_{rectal}
85	Rommel, SA; Early, GA; Matassa, KA; Pabst, DA; McLellan, WA	Venous structures associated with thermoregulation of phocid seal reproductive organs	The Anatomical Record	1995		X							X	X			
86	Boily, P; Lavigne, DM	Thermoregulation of juvenile grey seals, *Halichoerus grypus* , in air	Canadian Journal of Zoology	1996		X				X					X		STP
87	Kvadsheim, PH; Folkow, LP; Blix, AS	Thermal conductivity of minke whale blubber	Journal of Thermal Biology	1996		X								X			
88	Hansen, S; Lavigne, DM	Ontogeny of the thermal limits in the harbor seal (*Phoca vitulina*)	Physiological Zoology	1997		X				X					X		STP
89	Heyning, JE; Mead, JG	Thermoregulation in the mouths of feeding gray whales	Science	1997		X								X			
90	Hind, AT; Gurney, WS	The metabolic cost of swimming in marine homeotherms	The Journal of Experimental Biology	1997			X										
91	Kastelein, RA; Koene, P; Nieuwstraten, SH; Labberté, S	Skin surface temperature changes in a harbour porpoise (*Phocoena phocoena*) while on land	The Biology of the Harbour Porpoise	1997		X				X					X		IRT

Publication					Study Setting			Animal State						Biotelemetry		Notes
	Publication	Title		Year	Field	Lab	Modeling	Free-ranging	Trained	Captive	Restrained	Peri-mortem	Carcass	*Ex vivo*	Physiology	Behavior	
92	Kvadsheim, PH; Folkow, LP	Blubber and flipper heat transfer in harp seals	Acta Physiologica Scandinavica	1997		X	X		X					X			
93	Kvadsheim, PH; Gotaas, ARL; Folkow, LP; Blix, AS	An experimental validation of heat loss models for marine mammals	Journal of Theoretical Biology	1997		X	X		X			X		X			
94	Rosen, DAS; Renouf, D	Seasonal changes in blubber distribution in Atlantic harbor seals: indications of thermodynamic considerations	Marine Mammal Science	1997		X				X							
95	Hind, AT; Gurney, WSC	Are there thermoregulatory constraints on the timing of pupping for harbour seals?	Canadian Journal of Zoology	1998			X										
96	Koopman, HN	Topographical distribution of the blubber of harbor porpoises (*Phocoena phocoena*)	Journal of Mammalogy	1998		X							X				
97	Heath, ME; Ridgway, SH	How dolphins use their blubber to avoid heat stress during encounters with warm water	American Journal of Physiology-Regulatory, Integrative and Comparative Physiology	1999		X			X						X		HF, TC_{rectal}
98	Noren, DP; Williams, TM; Berry, P; Butler, E	Thermoregulation during swimming and diving in bottlenose dolphins, *Tursiops truncatus*	Journal of Comparative Physiology B	1999	X				X								diving physiology study, in water
99	Williams, TM; Noren, D; Berry, P; Estes, JA; Allison, C; Kirtland, J	The diving physiology of bottlenose dolphins (*Tursiops truncatus*). III. Thermoregulation at depth	Journal of Experimental Biology	1999	X				X						X	X	ECG, TDR, diving physiology study, in water
**2000-2009**																	
100	Boily, P; Kvadsheim, PH; Folkow, LP	Cutaneous heat flux models do not reliably predict metabolic rates of marine mammals	Journal of Theoretical Biology	2000		X	X				X		X	X			
101	Boyd, IL	Skin temperatures during free-ranging swimming and diving in Antarctic fur seals	Journal of Experimental Biology	2000	X		X	X							X	X	$\Delta TR_{skin-water}$, TDR, field component in water
102	Donohue, MJ; Costa, DP; Goebel, ME; Baker, JD	The ontogeny of metabolic rate and thermoregulatory capabilities of northern fur seal, *Callorhinus ursinus* , pups in air and water	Journal of Experimental Biology	2000	X						X						on land and in water
103	Mauck, B; Eysel, U; Dehnhardt, G	Selective heating of vibrissal follicles	Journal of Experimental Biology	2000		X			X						X		IRT (vibrissal pad)
104	Heyning, JE	Thermoregulation in feeding baleen whales: Morphological and physiological evidence	Aquatic Mammals	2001		X				X					X		IRT (tongue)
105	Jobsis, PD; Ponganis, PJ; Kooyman, GL	Effects of training on forced submersion responses in harbor seals	Journal of Experimental Biology	2001		X			X		X				X		ECG, muscle blood flow (via laser Doppler), diving physiology study
106	Rommel, SA; Pabst, DA; McLellan, WA	Functional morphology of venous structures associated with the male and female reproductive systems in Florida manatees (*Trichechus manatus latirostris*)	The Anatomical Record: An Official Publication of the American Association of Anatomists	2001		X								X			

Supplementary File

Publication					Study Setting			Animal State							Biotelemetry		Notes
	Publication	Title		Year	Field	Lab	Modeling	Free-ranging	Trained	Captive	Restrained	Peri-mortem	Carcass	*Ex vivo*	Physiology	Behavior	
107	Koopman, HN; Pabst, DA; Mclellan, WA; Dillaman, RM; Read, AJ	Changes in blubber distribution and morphology associated with starvation in the harbor porpoise (*Phocoena phocoena*): evidence for regional differences in blubber structure and function	Physiological and Biochemical Zoology	2002		X							X	X			
108	Kvadsheim, PH; Aarseth, JJ	Thermal function of phocid seal fur	Marine Mammal Science	2002		X								X			
109	Meagher, EM; McLellan, WA; Westgate, AJ; Wells, RS; Frierson Jr, D; Pabst, DA	The relationship between heat flow and vasculature in the dorsal fin of wild bottlenose dolphins *Tursiops truncatus*	Journal of Experimental Biology	2002	X						X				X		ECG, HF with integrated TC_{skin}, in water
110	Noren, DP	Thermoregulation of weaned northern elephant seal (*Mirounga angustirostris*) pups in air and water	Physiological and Biochemical Zoology	2002		X				X							
111	Twiss, SD; Wright, NC; Dunstone, N; Redman, P; Moss, S; Pomeroy, PP	Behavioral evidence of thermal stress from overheating in UK breeding gray seals	Marine Mammal Science	2002	X			X									in water
112	Mauck, B; Bilgmann, K; Jones, DD; Eysel, U; Dehnhardt, G	Thermal windows on the trunk of hauled-out seals: hot spots for thermoregulatory evaporation?	Journal of Experimental Biology	2003		X			X	X					X		IRT
113	Rommel, SA; Caplan, H	Vascular adaptations for heat conservation in the tail of Florida manatees (*Trichechus manatus latirostris*)	Journal of Anatomy	2003		X							X	X			
114	Rosen, DAS; Trites, AW	No evidence for bioenergetic interaction between digestion and thermoregulation in Steller sea lions *Eumetopias jubatus*	Physiological and Biochemical Zoology	2003		X			X								
115	Elsner, R; George, JC; O'hara, T	Vasomotor responses of isolated peripheral blood vessels from bowhead whales: thermoregulatory implications	Marine Mammal Science	2004		X								X			
116	Elsner, R; Meiselman, HJ; Baskurt, OK	Temperature-viscosity relations of bowhead whale blood: A possible mechanism for maintaining cold blood flow	Marine Mammal Science	2004		X								X			
117	Ortiz, RM; Worthy, GAJ	Could lower body fat mass contribute to cold-water susceptibility in calves of the West Indian manatee (*Trichechus manatus*)?	Marine Mammal Science	2004		X				X							
118	Rutishauser, MR; Costa, DP; Goebel, ME; Williams, TM	Ecological implications of body composition and thermal capabilities in young Antarctic fur seals (*Arctocephalus gazella*)	Physiological and Biochemical Zoology	2004	X		X				X						field component on land and in water
119	Samuel, AM; Worthy GAJ	Variability in fatty acid composition of bottlenose dolphin (*Tursiops truncatus*) blubber as a function of body site, season, and reproductive state	Canadian Journal of Zoology	2004		X								X			
120	Silva, RG	Assessment of body surface temperature in cetaceans: an iterative approach	Brazilian Journal of Biology	2004			X										
121	Struntz, DJ; McLellan, WA; Dillaman, RM; Blum, JE; Kucklick, JR; Pabst, DA	Blubber development in bottlenose dolphins (*Tursiops truncatus*)	Journal of Morphology	2004		X							X	X			

Supplementary File

Publication					Study Setting			Animal State							Biotelemetry		Notes
	Publication	Title		Year	Field	Lab	Modeling	Free-ranging	Trained	Captive	Restrained	Peri-mortem	Carcass	*Ex vivo*	Physiology	Behavior	
122	Bossart, GD; Meisner, RA; Rommel, SA; Ghim, S-J; Jenson, AB	Pathological features of the Florida manatee cold stress syndrome	Aquatic Mammals	2005		X							X				
123	Dunkin, RC; McLellan, WA; Blum, JE; Pabst, DA	The ontogenetic changes in the thermal properties of blubber from Atlantic bottlenose dolphin *Tursiops truncatus*	Journal of Experimental Biology	2005		X							X	X			
124	Harding, KC; Fujiwara, M; Axberg, Y; Härkönen, T	Mass-dependent energetics and survival in harbour seal pups	Functional Ecology	2005	X		X				X						field component on land
125	Kvadsheim, PH; Folkow, LP; Blix, AS	Inhibition of shivering in hypothermic seals during diving	American Journal of Physiology-Regulatory, Integrative and Comparative Physiology	2005		X			X		X				X		EMG, TC_{brain}, TC_{rectal}, diving physiology study
126	McCafferty, DJ; Moss, S; Bennett, K; Pomeroy, PP	Factors influencing the radiative surface temperature of grey seal (*Halichoerus grypus*) pups during early and late lactation	Journal of Comparative Physiology B	2005	X						X				X		IRT (rectal), on land
127	Rotherham, LS; van Der Merwe, M; Bester, MN; Oosthuizen, WH	Morphology and distribution of sweat glands in the Cape fur seal, *Arctocephalus pusillus pusillus* (Carnivora: Otariidae)	Australian Journal of Zoology	2005		X								X			
128	Thornton, SJ; Hochachka, PW; Crocker, DE; Costa, DP; LeBoeuf, BJ; Spielman, DM; Pelc, NJ	Stroke volume and cardiac output in juvenile elephant seals during forced dives	Journal of Experimental Biology	2005		X					X						diving physiology
129	Willis, K; Horning, M	A novel approach to measuring heat flux in swimming animals	Journal of Experimental Marine Biology and Ecology	2005	X	X		X	X						X		HF with integrated TC_{skin}, field component in water
130	Willis, K; Horning, M; Rosen, DAS; Trites, AW	Spatial variation of heat flux in Steller sea lions: evidence for consistent avenues of heat exchange along the body trunk	Journal of Experimental Marine Biology and Ecology	2005		X	X		X						X	X	HF with integrated TR_{skin}, swim speed
131	Beentjes, MP	Behavioral thermoregulation of the New Zealand sea lion (*Phocarctos hookeri*)	Marine Mammal Science	2006	X			X					X				
132	Ponganis, PJ; Stockard, T Knower; Levenson, DH; Berg, L; Baranov, EA	Cardiac output and muscle blood flow during rest-associated apneas of elephant seals	Comparative Biochemistry and Physiology Part A: Molecular & Integrative Physiology	2006		X				X					X		muscle blood flow (via laser Doppler), ECG, thermistor-tipped catheter (T_{blood})
133	Koopman, HN	Phylogenetic, ecological, and ontogenetic factors influencing the biochemical structure of the blubber of odontocetes	Marine Biology	2007		X								X			
134	Mellish, JE; Horning, M; York, AE	Seasonal and spatial blubber depth changes in captive harbor seals (*Phoca vitulina*) and Steller's sea lions (*Eumetopias jubatus*)	Journal of Mammalogy	2007		X			X								
135	Werth, AJ	Adaptations of the cetacean hyolingual apparatus for aquatic feeding and thermoregulation	The Anatomical Record: Advances in Integrative Anatomy and Evolutionary Biology	2007		X								X			

Supplementary File

Publication					Study Setting			Animal State							Biotelemetry		Notes
	Publication	Title		Year	Field	Lab	Modeling	Free-ranging	Trained	Captive	Restrained	Peri-mortem	Carcass	*Ex vivo*	Physiology	Behavior	
136	Westgate, AJ; McLellan, WA; Wells, RS; Scott, MD; Meagher, EM; Pabst, DA	A new device to remotely measure heat flux and skin temperature from free-swimming dolphins	Journal of Experimental Marine Biology and Ecology	2007	X			X							X	X	HF with integrated TR_{skin}, swim velocity, TDR, VHF, in water
137	Hasselberg, BA; Thomas, JA	Using an infrared temperature sensor to study microhabitat selection in captive California Sea Lions (*Zalophus californianus*)	Aquatic Mammals	2008		X				X					X		IRT
138	Meagher, EM; McLellan, WA; Westgate, AJ; Wells, RS; Blum, JE; Pabst, DA	Seasonal patterns of heat loss in wild bottlenose dolphins (*Tursiops truncatus*)	Journal of Comparative Physiology B	2008	X						X				X		HF with integrated TC_{skin}, TC_{rectal}, in water
139	Montie, EW; Garvin, SR; Fair, PA; Bossart, GD; Mitchum, GB; McFee, WE; Speakman, T; Starczak, VR; Hahn, ME	Blubber morphology in wild bottlenose dolphins (*Tursiops truncatus*) from the Southeastern United States: influence of geographic location, age class, and reproductive state	Journal of Morphology	2008		X								X			
140	Noren, SR; Pearson, LE; Davis, J; Trumble, SJ; Kanatous, SB	Different thermoregulatory strategies in nearly weaned pup, yearling, and adult Weddell seals (*Leptonychotes weddelli*)	Physiological and Biochemical Zoology	2008	X						X						on land
141	Ponganis, PJ; Kreutzer, U; Stockard, TK; Lin, P-C; Sailasuta, N; Tran, T-K; Hurd, R; Jue, T	Blood flow and metabolic regulation in seal muscle during apnea	Journal of Experimental Biology	2008		X			X						X		muscle blood flow (via laser Doppler)
142	Yeates, LC; Houser, DS	Thermal tolerance in bottlenose dolphins (*Tursiops truncatus*)	Journal of Experimental Biology	2008		X			X								
143	Castellini, MA; Trumble, SJ; Mau, TL; Yochem, PK; Stewart, BS; Koski, MA	Body and blubber relationships in Antarctic pack ice seals: implications for blubber depth patterns	Physiological and Biochemical Zoology	2009	X						X						on land
144	Hammill, MO; Ryg, M; Chabot, D	Seasonal changes in energy requirements of harp seals	Journal of Northwest Atlantic Fishery Science	2009			X										
145	Liwanag, HEM; Williams, TM; Costa, DP; Kanatous, SB; Davis, RW; Boyd, IL	The effects of water temperature on the energetic costs of juvenile and adult California sea lions (Zalophus californianus): the importance of skeletal muscle thermogenesis for thermal balance	Journal of Experimental Biology	2009		X			X						X		STP
146	Noren, SR; Wells, RS	Blubber deposition during ontogeny in free-ranging bottlenose dolphins: balancing disparate roles of insulation and locomotion	Journal of Mammalogy	2009	X						X						in water
**2010-2019**																	
147	Barbieri, MM; McLellan, WA; Wells, RS; Blum, JE; Hofmann, S; Gannon, J; Pabst, DA	Using infrared thermography to assess seasonal trends in dorsal fin surface temperatures of free-swimming bottlenose dolphins (*Tursiops truncatus*) in Sarasota Bay, Florida	Marine Mammal Science	2010	X			X							X		IRT (dorsal fin), in water
148	Blix, AS; Walløe, L; Messelt, EB; Folkow, LP	Selective brain cooling and its vascular basis in diving seals	Journal of Experimental Biology	2010		X					X			X	X		TC_{brain}, TC_{blood}, TC_{muscle}, TC_{rectal}, diving physiology study

Supplementary File

Publication					Study Setting			Animal State							Biotelemetry		Notes
	Publication	Title		Year	Field	Lab	Modeling	Free-ranging	Trained	Captive	Restrained	Peri-mortem	Carcass	*Ex vivo*	Physiology	Behavior	
149	Liwanag, HEM	Energetic costs and thermoregulation in northern fur seal (*Callorhinus ursinus*) pups: the importance of behavioral strategies for thermal balance in furred marine mammals	Physiological and Biochemical Zoology	2010		X			X	X							
150	Meir, JU; Ponganis, PJ	Blood temperature profiles of diving elephant seals	Physiological and Biochemical Zoology	2010	X			X							X	X	P_{O2} electrode, TR_{blood}, TDR, SAT, VHF, diving physiology study, in water
151	Nienaber, J; Thomton, H; Horning, M; Polasek, L; Mellish, J-A	Surface temperature patterns in seals and sea lions: a validation of temporal and spatial consistency	Journal of Thermal Biology	2010		X			X						X		IRT
152	Norris, AL; Houser, DS; Crocker, DE	Environment and activity affect skin temperature in breeding adult male elephant seals (*Mirounga angustirostris*)	Journal of Experimental Biology	2010	X			X							X		IRT, on land
153	Houser, DS; Yeates, LC; Crocker, DE	Cold stress induces an adrenocortical response in bottlenose dolphins (*Tursiops truncatus*)	Journal of Zoo and Wildlife Medicine	2011		X			X	X							
154	Bagge, LE; Koopman, HN; Rommel, SA; McLellan, WA; Pabst, DA	Lipid class and depth-specific thermal properties in the blubber of the short-finned pilot whale and the pygmy sperm whale	Journal of Experimental Biology	2012		X								X			
155	Durban, JW; Pitman, RL	Antarctic killer whales make rapid, round-trip movements to subtropical waters: evidence for physiological maintenance migrations?	Biology Letters	2012	X			X								X	SAT, in water
156	Erdsack, N; Hanke, FD; Dehnhardt, G; Hanke, W	Control and amount of heat dissipation through thermal windows in harbor seals (*Phoca vitulina*)	Journal of Thermal Biology	2012		X	X	X	X						X		IRT
157	Khamas, WA; Smodlaka, H; Leach-Robinson, J; Palmer, L	Skin histology and its role in heat dissipation in three pinniped species	Acta Veterinaria Scandinavica	2012		X								X			
158	Liwanag, HEM; Berta, A; Costa, DP; Abney, M; Williams, TM	Morphological and thermal properties of mammalian insulation: the evolution of fur for aquatic living	Biological Journal of the Linnean Society	2012		X								X			
159	Liwanag, HEM; Berta, A; Costa, DP; Budge, SM; Williams, TM	Morphological and thermal properties of mammalian insulation: the evolutionary transition to blubber in pinnipeds	Biological Journal of the Linnean Society	2012		X								X			
160	McClelland, SJ; Gay, M; Pabst, DA; Dillaman, R; Westgate, AJ; Koopman, HN	Microvascular patterns in the blubber of shallow and deep diving odontocetes	Journal of Morphology	2012		X								X			
161	Mellish, J; Nienaber, J; Polasek, L; Horning, M	Beneath the surface: Profiling blubber depth in pinnipeds with infrared imaging	Journal of Thermal Biology	2012		X			X						X		IRT
162	Paterson, W; Sparling, CE; Thompson, D; Pomeroy, PP; Currie, JI; McCafferty, DJ	Seals like it hot: Changes in surface temperature of harbour seals (*Phoca vitulina*) from late pregnancy to moult	Journal of Thermal Biology	2012		X				X					X		IRT

Supplementary File

Publication					Study Setting			Animal State							Biotelemetry		Notes
	Publication	Title		Year	Field	Lab	Modeling	Free-ranging	Trained	Captive	Restrained	Peri-mortem	Carcass	*Ex vivo*	Physiology	Behavior	
163	Erdsack, N; Dehnhardt, G; Hanke, W	Coping with heat: function of the natal coat of Cape fur seal (*Arctocephalus pusillus pusillus*) pups in maintaining core body temperature	PLoS ONE	2013		X				X	X				X		IRT
164	Ford Jr, TJ; Werth, AJ; George, JC	An intraoral thermoregulatory organ in the bowhead whale (*Balaena mysticetus*), the corpus cavernosum maxillaris	The Anatomical Record	2013		X								X	X		IRT (oral cavity)
165	Rodríguez-Prieto, V; Rubio-García, A; Melero, M; García, D; Sánchez-Vizcaíno, JM	Identification of the pattern of appearance and development of thermal windows in the skin of juvenile Pacific walruses (*Odobenus rosmarus divergens*) in a controlled environment	Marine Mammal Science	2013		X			X						X		IRT
166	Dalton, AJM; Rosen, DAS; Trites, AW	Broad thermal capacity facilitates the primarily pelagic existence of northern fur seals (*Callorhinus ursinus*)	Marine Mammal Science	2014		X			X								
167	Erdsack, N; Dehnhardt, G; Hanke, W	Thermoregulation of the vibrissal system in harbor seals (*Phoca vitulina*) and Cape fur seals (*Arctocephalus pusillus pusillus*)	Journal of Experimental Marine Biology and Ecology	2014	X				X						X		IRT (vibrissal pad), on land
168	Garlepp, L; Logan, M; Kirkwood, R	Behavioral responses of Australian fur seals (*Arctocephalus pusillus doriferus*) to environmental variations	Marine Mammal Science	2014	X			X									on land
169	Horgan, P; Booth, D; Nichols, C; Lanyon, JM	Insulative capacity of the integument of the dugong (*Dugong dugon*): thermal conductivity, conductance and resistance measured by in vitro heat flux	Marine biology	2014		X								X			
170	Liwanag, HEM; Oraze, J; Costa, DP; Williams, TM	Thermal benefits of aggregation in a large marine endotherm: huddling in California sea lions	Journal of Zoology	2014	X			X							X		IRT, on land
171	Pearson, LE; Liwanag, HEM; Hammill, MO; Burns, JM	Shifts in thermoregulatory strategy during ontogeny in harp seals (*Pagophilus groenlandicus*)	Journal of Thermal Biology	2014		X							X	X			
172	Pearson, LE; Liwanag, HEM; Hammill, MO; Burns, JM	To each its own: Thermoregulatory strategy varies among neonatal polar phocids	Comparative Biochemistry and Physiology Part A: Molecular & Integrative Physiology	2014		X							X	X			
173	Rosen, DAS; Trites, AW	Thermal limits in young northern fur seals, *Callorhinus ursinus*	Marine Mammal Science	2014		X			X								
174	Ball, HC; Stavarz, M; Oldaker, J; Usip, S; Londraville, RL; George, JC; Thewissen, JGM; Duff, RJ	Seasonal and ontogenetic variation in subcutaneous adipose of the bowhead whale (*Balaena mysticetus*)	The Anatomical Record	2015		X								X			
175	Ekdale, EG; Kienle, SS	Passive Restriction of Blood Flow and Counter-Current Heat Exchange Via Lingual Retia in the Tongue of a Neonatal Gray Whale E schrichtius robustus (Cetacea, Mysticeti)	The Anatomical Record	2015		X							X				

Publication					Study Setting			Animal State							Biotelemetry		Notes
	Publication	Title		Year	Field	Lab	Modeling	Free-ranging	Trained	Captive	Restrained	Peri-mortem	Carcass	*Ex vivo*	Physiology	Behavior	
176	Erdsack, N; Dehnhardt, G; Witt, M; Wree, A; Siebert, U; Hanke, W	Unique fur and skin structure in harbour seals (*Phoca vitulina*)—thermal insulation, drag reduction, or both?	Journal of the Royal Society Interface	2015		X								X			
177	Gmuca, NV; Pearson, LE; Burns, JM; Liwanag, HEM	The fat and the furriest: morphological changes in harp seal fur with ontogeny	Physiological and Biochemical Zoology	2015		X								X			
178	Gómez-Campos, E; Borrell, A; Correas, J; Aguilar, A	Topographical variation in lipid content and morphological structure of the blubber in the striped dolphin	Scientia Marina	2015		X								X			
179	Hashimoto, O; Ohtsuki, H; Kakizaki, T; Amou, K; Sato, R; Doi, S; Kobayashi, S; Matsuda, A; Sugiyama, M; Funaba, M	Brown adipose tissue in cetacean blubber	PLoS ONE	2015		X								X			
180	Hindle, AG; Horning, M; Mellish, JAE	Estimating total body heat dissipation in air and water from skin surface heat flux telemetry in Weddell seals	Animal Biotelemetry	2015	X		X	X							X	X	STP, HF sensor with integrated thermistor (T_{skin}), IRT, TDR, accelerometer, SAT, VHF, field component on land and in water
181	Melero, M; Rodríguez-Prieto, V; Rubio-García, A; García-Párraga, D; Sánchez-Vizcaíno, JM	Thermal reference points as an index for monitoring body temperature in marine mammals	BMC Research Notes	2015		X			X						X		IRT
182	Mellish, J-A; Hindle, A; Skinner, J; Horning, M	Heat loss in air of an Antarctic marine mammal, the Weddell seal	Journal of Comparative Physiology B	2015	X		X	X			X				X		IRT, field component on land
183	Noren, SR; Udevitz, MS; Triggs, L; Paschke, J; Oland, L; Jay, CV	Identifying a reliable blubber measurement site to assess body condition in a marine mammal with topographically variable blubber, the Pacific walrus	Marine Mammal Science	2015		X			X								
184	Sakurai, Y; Okamatsu-Ogura, Y; Saito, M; Kimura, K; Nakao, R; Ohnuma, A; Kobayashi, M	Brown adipose tissue expresses uncoupling protein 1 in newborn harbor seals (*Phoca vitulina*)	Marine Mammal Science	2015		X								X			
185	Zeng, X; Ji, J; Hao, Y; Wang, D	Topographical distribution of blubber in finless porpoises (*Neophocaena asiaeorientalis sunameri*): a result from adapting to living in coastal waters	Zoological Studies	2015		X							X	X			
186	Codde, SA; Allen, SG; Houser, DS; Crocker, DE	Effects of environmental variables on surface temperature of breeding adult female northern elephant seals, *Mirounga angustirostris* , and pups	Journal of Thermal Biology	2016	X			X							X		IRT, on land
187	Cornick, LA; Quakenbush, LT; Norman, SA; Pasi, C; Maslyk, P; Burek, DA; Goertz, CEC; Hobbs, RC	Seasonal and developmental differences in blubber stores of beluga whales in Bristol Bay, Alaska using high-resolution ultrasound	Journal of Mammalogy	2016	X						X		X				in water

Supplementary File

Publication					Study Setting			Animal State							Biotelemetry		Notes
	Publication	Title		Year	Field	Lab	Modeling	Free-ranging	Trained	Captive	Restrained	Peri-mortem	Carcass	*Ex vivo*	Physiology	Behavior	
188	Beltran, RS; Testa, JW; Burns, JM	An agent-based bioenergetics model for predicting impacts of environmental change on a top marine predator, the Weddell seal	Ecological Modelling	2017			X										
189	Ladds, MA; Slip, DJ; Harcourt, RG	Intrinsic and extrinsic influences on standard metabolic rates of three species of Australian otariid	Conservation physiology	2017		X			X								
190	Sharma, N; Liwanag, HEM	The effects of submergence on the thermal function of pinniped fur	Marine Mammal Science	2017		X								X			
191	Singleton, EM; McLellan, WA; Koopman, HN; Pokorny, A; Scharf, FS; Pabst, DA	Lipid composition and thermal properties of the blubber of Gervais' beaked whale (*Mesoplodon europaeus*) across ontogeny	Marine Mammal Science	2017		X								X			
192	Chaise, LL; Prinet, I; Toscani, C; Gallon, SL; Paterson, W; McCafferty, DJ; Théry, M; Ancel, A; Gilbert, C	Local weather and body condition influence habitat use and movements on land of molting female southern elephant seals (*Mirounga leonina*)	Ecology and Evolution	2018	X			X								X	GPS, VHF, on land
193	Erdsack, N; Phillips, SRM; Rommel, SA; Pabst, DA; McLellan, WA; Reynolds, JE	Heat flux in manatees: an individual matter and a novel approach to assess and monitor the thermal state of Florida manatees (*Trichechus manatus latirostris*)	Journal of Comparative Physiology B	2018		X			X								
194	García-Aguilar, AC; Turrent, C; Elorriaga-Verplancken, FR; Arias-Del-Razo, A; Schramm, Y	Climate change and the northern elephant seal (*Mirounga angustirostris*) population in Baja California, Mexico	PLoS ONE	2018	X			X									on land
195	Krmpotic, CM; Loza, CM; Negrete, J; Scarano, AC; Carlini, AA; Guerrero, A; Barbeito, CG	Integument in Antarctic seals: a comparative study and its relation to extreme environments	Acta Zoologica	2018		X								X			
196	Rojano-Doñate, L; McDonald, BI; Wisniewska, DM; Johnson, M; Teilmann, J; Wahlberg, M; Højer-Kristensen, J; Madsen, PT	High field metabolic rates of wild harbour porpoises	Journal of Experimental Biology	2018	X	X		X	X		X					X	TDR, accelerometer, SAT, VHF, field component in water
197	Zeh, DR; Heupel, MR; Hamann, M; Jones, R; Limpus, CJ; Marsh, H	Evidence of behavioural thermoregulation by dugongs at the high latitude limit to their range in eastern Australia	Journal of Experimental Marine Biology and Ecology	2018	X			X								X	SAT, GPS, acoustic transmitter (depth & T_{water}), in water
198	Adamczak, SK; Pabst, DA; McLellan, WA; Thorne, LH	Using 3D Models to Improve Estimates of Marine Mammal Size and External Morphology	Frontiers in Marine Science	2019			X						X				
199	Chaise, LL; McCafferty, DJ; Krellenstein, A; Gallon, SL; Paterson, WD; Théry, M; Ancel, A; Gilbert, C	Environmental and physiological determinants of huddling behavior of molting female southern elephant seals (*Mirounga leonina*)	Physiology & Behavior	2019	X			X							X	X	STP, IRT, VHF, TDR, on land

Publication					Study Setting			Animal State						Biotelemetry		Notes
	Publication	Title		Year	Field	Lab	Modeling	Free-ranging	Trained	Captive	Restrained	Peri-mortem	Carcass	*Ex vivo*	Physiology	Behavior	
200	Guerrero, AI; Rogers, TL	From low to high latitudes: changes in fatty acid desaturation in mammalian fat tissue suggest a thermoregulatory role	BMC Evolutionary Biology	2019		X								X			
201	Ji, J; Nabi, G; Zeng, X; Hao, Y; Wang, D	Histological variation in blubber morphology of the endangered east asian finless porpoise (*Neophocaena asiaeorientalis sunameri*) with ontogeny and reproductive states	Zoological Studies	2019		X							X	X			
202	Kastelein, RA; Helder-Hoek, L; Jennings, N; van Kester, R; Huisman, R	Reduction in Body Mass and Blubber Thickness of Harbor Porpoises (*Phocoena phocoena*) Due to Near-Fasting for 24 Hours in Four Seasons	Aquatic Mammals	2019		X			X								
203	Lonati, GL; Singleton, EM; Phelps, CE; Koopman, HN; Pabst, DA	The density of odontocete integument depends on blubber lipid composition and temperature	Marine Mammal Science	2019		X								X			
204	Pearson, LE; Weitzner, EL; Burns, JM; Hammill, MO; Liwanag, HEM	From ice to ocean: changes in the thermal function of harp seal pelt with ontogeny	Journal of Comparative Physiology B	2019		X	X							X			
205	Pitman, RL; Durban, JW; Joyce, T; Fearnbach, H; Panigada, S; Lauriano, G	Skin in the game: Epidermal molt as a driver of long-distance migration in whales	Marine Mammal Science	2019	X			X								X	SAT, TDR, in water
206	Plön, S; Frainer, G; Wedderburn-Maxwell, A; Cliff, G; Huggenberger, S	Dorsal fin and hump vascular anatomy in the Indo-Pacific humpback dolphin (*Sousa plumbea*) and the Indo-Pacific bottlenose dolphin (*Tursiops aduncus*)	Marine Mammal Science	2019		X								X			
207	Tuneu-Corral, C; Szteren, D; Cassini, MH	Living on the edge: thermoregulatory behaviour of South American sea lions, *Otaria flavescens* , at the northern limit of their Atlantic distribution	Acta Ethologica	2019	X			X									on land
**2020-2021**																	
208	Adamczak, SK; Pabst, DA; McLellan, WA; Thorne, LH	Do bigger bodies require bigger radiators? Insights into thermal ecology from closely related marine mammal species and implications for ecogeographic rules	Journal of Biogeography	2020			X						X				not included in figure
209	Adamczak, SK; McLellan, WA; Read, AJ; Wolfe, CLP; Thorne, LH	The impact of temperature at depth on estimates of thermal habitat for short-finned pilot whales	Marine Mammal Science	2020	X			X								X	not included in figure, TDR
210	Chambault, P; Tervo, OM; Garde, E; Hansen, RG; Blackwell, SB; Williams, TM; Dietz, R; Albertsen, CM; Laidre, KL; Nielsen, NH	The impact of rising sea temperatures on an Arctic top predator, the narwhal	Scientific Reports	2020	X			X								X	not included in figure, SAT, GPS, in water

Supplementary File

Publication					Study Setting			Animal State							Biotelemetry		Notes
	Publication	Title		Year	Field	Lab	Modeling	Free-ranging	Trained	Captive	Restrained	Peri-mortem	Carcass	*Ex vivo*	Physiology	Behavior	
211	da Silva, AP; Machado, ASD; Le Bas, AE; Silva, RG; dos Anjos Silva, E; Hernandez-Blazquez, FJ	The skin structures and their role in the thermoregulation of the South American fur seal (*Arctocephalus australis*)	The Anatomical Record	2020		X								X			not included in figure
212	Haase, CG; Fletcher Jr, RJ; Slone, DH; Reid, JP; Butler, SM	Traveling to thermal refuges during stressful temperatures leads to foraging constraints in a central-place forager	Journal of Mammalogy	2020	X			X								X	not included in figure, SAT, GPS, in water
213	Heide-Jørgensen, MP; Blackwell, SB; Williams, TM; Sinding, MHS; Skovrind, M; Tervo, OM; Garde, E; Hansen, RG; Nielsen, NH; Ngô, MC	Some like it cold: Temperature-dependent habitat selection by narwhals	Ecology and evolution	2020	X			X								X	not included in figure, TDR, CTD, SAT, GPS, in water
214	Walcott, SM; Kirkham, AL; Burns, JM	Thermoregulatory costs in molting Antarctic Weddell seals: impacts of physiological and environmental conditions	Conservation physiology	2020	X						X				X		not included in figure, IRT, on land
215	Guerrero, AI; Rogers, TL; Sepúlveda, M	Conditions influencing the appearance of thermal windows and the distribution of surface temperature in hauled-out southern elephant seals	Conservation Physiology	2021	X			X							X		not included in figure, IRT, in water
216	John, JS; Thometz, NM; Boerner, K; Denum, L; Kendall, TL; Richter, BP; Gaspard, JC; Williams, TM	Metabolic tradeoffs in tropical and subtropical marine mammals-Unique maintenance and locomotion costs in West Indian manatees and Hawaiian monk seals	Journal of Experimental Biology	2021		X			X							X	not included in figure, accelerometry, on land
217	Sumich, JL	Why Baja? A bioenergetic model for comparing metabolic rates and thermoregulatory costs of gray whale calves (*Eschrichtius robustus*)	Marine Mammal Science	2021			X										not included in figure
218	Wright, T; Davis, RW; Pearson, HC; Murray, M; Sheffield-Moore, M	Skeletal muscle thermogenesis enables aquatic life in the smallest marine mammal	Science	2021		X								X			not included in figure

References

1. Scholander PF, Irving L, Grinnell SW. On the Temperature and Metabolism of the seal during diving. *J Cell Comp Physiol* 1942;**19**:67–78.

2. Parry DA. The Structure of Whale Blubber, and a Discussion of its Thermal Properties. *Q J Microsc Sci* 1949;**90**:13–25.

3. Scholander PF, Schevill WE. Counter-Current Vascular Heat Exchange in the Fins of Whales. *J Appl Physiol* 1955;**8**:279–82.

4. Bartholomew GA, Wilke F. Body Temperature in the Northern Fur Seal, Callorhinus ursinus. *J Mammal* 1956;**37**:327–37.

5. Irving L, Hart JS. The metabolism and insulation of seals as bare-skinned mammals in cold water. *Can J Zool* 1957;**35**:497–511.

6. Hart JS, Irving L. The energetics of harbor seals in air and in water with special consideration of seasonal changes. *Can J Zool* 1959;**37**:447–57.

7. Irving L, Peyton LJ, Bahn CH *et al.* Regulation of Temperature in Fur Seals. *Physiol Zool* 1962;**35**:275–84.

8. Bryden MM. Insulating capacity of the subcutaneous fat of the Southern elephant seal. *Nature* 1964:1299–300.

9. Ling JK. Functional significance of sweat glands and sebaceous glands in seals. *Nature* 1965;**208**:560–2.

10. Ling JK. The skin and hair of the Southern Elephant Seal Mirounga leonina (L.) III. morphology of the adult integument. *Aust J Zool* 1968;**16**:629–45.

11. Tarasoff FJ, Fisher HD. Anatomy of the hind flippers of two species of seals with reference to thermoregulation. *Can J Zool* 1970;**48**:821–9.

12. Hampton IFG, Whittow GC, Szekerczes J *et al.* Heat transfer and body temperature in the Atlantic bottlenosed dolphin, Tursiops truncatus. *Int J Biometerology* 1971;**15**:247–53.

13. McGinnis SM, Southworth TP. Thermoregulation in the northern elephant seal, Mirounga angustirostris. *Comp Biochem Physiol* 1971;**40A**:893–8.

14. White FN, Odell DK. Thermoregulatory Behavior of the Northern Elephant Seal, Mirounga angustirostris. *J Mammal* 1971;**52**:758–74.

15. McGinnis SM, Whittow GC, Ohata CA *et al.* Body heat dissipation and conservation in two species of dolphins. *Comp Biochem Physiol* 1972;**43**:417–23.

16. Ohata CA, Matsuura DT, Whittow GC *et al.* Diurnal Rhythm of Body Temperature in the Hawaiian Monk Seal (Monachus schauinslandi). *Pacific Sci* 1972;**26**:117–20.

17. Whittow GC, Matsuura DT, Lin YC. Temperature Regulation in the California Sea Lion (Zalophus californianus). *Physiol Zool* 1972;**45**:68–77.

18. Gentry RL. Thermoregulatory Behavior of Eared Seals. *Behaviour* 1973;**46**:73–93.

19. Matsuura DT, Whittow GC. Evaporative heat loss in the California sea lion and harbor seal. *Comp Biochem Physiol* 1974;**48**:20.

20. Morrison P, Rosenmann M, Estes JA. Metabolism and Thermoregulation in the Sea Otter. *Physiol Zool* 1974;**47**:218–29.

21. Odell DK. Behavioral thermoregulation in the California Sea Lion. *Behav Biol* 1974;**10**:231–7.

22. Ohata CA, Whittow GC. Conductive heat loss to sand in California sea lions and a harbor seal. *Comp Biochem Physiol* 1974;**47**:23–6.

23. Whittow GC, Hampton IFG, Matsuura DT *et al.* Body Temperature of Three Species of Whales. *J Mammal* 1974;**55**:653–6.

24. Luecke RH, Natarajan V, South FE. A mathematical biothermal model of the california sea lion. *J Therm Biol* 1975;**1**:35–45.

25. McGinnis SM. Peripheral Heat Exchange in Phocids. *Rapp P-v Reun Cons int Explor Mer* 1975;**169**:481–6.

26. Miller K, Irving L. Metabolism and temperature regulation in young harbor seals Phoca vitulina richardi. *Am J Physiol* 1975;**229**:506–11.

27. Molyneux AGS, Bryden MM. Arteriovenous Anastomoses in the Skin of the Weddell Seal, Leptonychotes weddelli. *Science (80-)* 1975;**189**:1100–2.

28. Whittow GC, Szekerczes J, Kridler E *et al.* Skin Structure of the Hawaiian Monk Seal (Monachus schauinslandi). *Pacific Sci* 1975;**29**:153–7.

29. Hampton IFG, Whittow GC. Body temperature and heat exchange in the Hawaiian spinner dolphin, Stenella longirostris. *Comp Biochem Physiol* 1976;**55A**:195–7.

30. Miller K, Rosenmann M, Morrison P. Oxygen uptake and temperature regulation of young harbor seals (Phoca vitulina richardi) in water. *Comp Biochem Physiol Part A Physiol* 1976;**54**:105–7.

31. Hammel HT, Elsner RW, Heller HC *et al.* Thermoregulatory responses to altering hypothalamic temperature in the harbor seal. *Am J Physiol Regul Integr Comp Physiol* 1977;**232**:R18–26.

32. Ohata CA, Miller LK. Northern fur seal thermoregulation: thermal responses to forced activity on land. *J Therm Biol* 1977;**2**:135–40.

33. Ohata CA., Miller L. K. Some Temperature Responses of Northern Fur Seal (Callorhinus ursinus) Pups. *J Mammal* 1977;**58**:438–40.

34. Ohata CA, Miller LK, Kajimura H. Northern fur seal thermoregulation: Thermal responses to pelagic conditions. *J Therm Biol* 1977;**2**:135–40.

35. Bryden MM, Molyneux GS. Arteriovenous anastomoses in the skin of seals: II. The California Sea Lion Zalophus californianus and the Northern Fur Seal Callorhinus ursinus (Pinnipedia: Otariidae). *Anat Rec* 1978;**96**:285–300.

36. Molyneux GS, Bryden MM. Arteriovenous Anastomoses in the Skin of Seals: I. The Weddell Seal Leptonychotes weddelli and the Elephant Seal Mirounga leonina (Pinnipedia: Phocidae). *Anat Rec* 1978;**191**:239–51.

37. Øritsland NA, Ronald K. Aspects of temperature regulation in harp seal pups evaluated by in vivo experiments and computer simulations. *Acta Physiol Scand* 1978;**103**:263–9.

38. Blix AS, Grav HJ, Ronald K. Some aspects of temperature regulation in newborn harp seal pups. *Am J Physiol* 1979;**236**:R188–97.

39. Blix AS, Miller LK, Keyes MC *et al.* Newborn northern fur seals (Cullorhinus zlrsinus)--do they suffer from cold? *Am J Physiol* 1979;**236**:R322-327.

40. Gallivan GJ, Ronald K. Temperature regulation in freely diving harp seals (Phoca groenlandica). *Can J Zool* 1979;**57**:2256–63.

41. Zapol WM, Liggins GC, Schneider RC *et al.* Regional blood flow during simulated diving in the conscious Weddell seal. *J Appl Physiol* 1979;**47**:968–73.

42. Kooyman GL, Wahrenbrock EA, Castellini MA *et al.* Aerobic and anaerobic metabolism during voluntary diving in Weddell seals: Evidence of preferred pathways from blood chemsitry and behavior. *J Comp Physiol B* 1980;**138**:335–46.

43. Costa DP, Kooyman GL. Oxygen consumption, thermoregulation, and the effect of fur oiling and washing on the sea otter, Enhydra lutris. *Can J Zool* 1982;**60**:2761–7.

44. Blix AS, Fay FH, Ronald K. On testicular cooling in phocid seals. *Polar Res* 1983;**1**:231–3.

45. Gallivan GJ, Best RC, Kanwisher JW. Temperature Regulation in the Amazonian Manatee Trichechus inunguis. *Physiol Zool* 1983;**56**:255–62.

46. Irvine AB. Manatee Metabolism and Its Influence on Distribution in Florida. *Biol Conserv* 1983;**25**:315–34.

47. Costa DP, Kooyman GL. Contribution of Specific Dynamic Action to Heat Balance and Thermoregulation in the Sea Otter Enhydra lutris. *Physiol Zool* 1984;**57**:199–203.

48. Brodie P, Paasche A. Thermoregulation and energetics of fin and sei whales based on postmortem, stratified temperature measurements. *Can J Zool* 1985;**63**:2267–9.

49. Limberger D, Trillmich F, Biebach H *et al.* Temperature regulation and microhabitat choice by free-ranging Galapagos fur seal pups (Arctocephalus galapagoensis). *Oecologia* 1986;**69**:53–9.

50. Yasui WY, Gaskin DE. Energy budget of a small cetacean, the harbour porpoise, phocoena phocoena (L.). *Ophelia* 1986;**25**:183–97.

51. Folkow LP, Schytte Blix A. Nasal heat and water exchange in gray seals. *Am J Physiol* 1987;**253**:R883–9.

52. Hill RD, Schneider RC, Liggins GC *et al.* Heart rate and body temperature during free diving of Weddell seals. *Am Physiol Soc* 1987:R344–51.

53. Thompson SD., Ono KA., Oftedal OT. *et al.* Thermoregulation and Resting Metabolic Rate of California Sea Lion (Zalophus californianus) Pups. *Physiol Zool* 1987;**60**:730–6.

54. Campagna C, Le Boeuf BJ. Thermoregulatory behaviour of southern sea lions and its effect on mating strategies. *Behaviour* 1988;**107**:72–90.

55. Ryg M, Smith TG, Øritsland NA. Thermal significance of the topographical distribution of blubber in ringed seals (Phoca hispida). *Can J Fish Aquat Sci* 1988;**45**:985–92.

56. Folkow LP, Schytte Blix A. Thermoregulatory control of expired air temperature in diving harp seals. *Am J Physiol - Regul Integr Comp Physiol* 1989;**257**:306–10.

57. Kasting NW, Adderley SAL, Safford T *et al.* Thermoregulation in Beluga (Delphinapterus leucas) and Killer (Orcinus orca) Whales. *Physiol Zool* 1989;**62**:687–701.

58. Doidge DW. Integumentary heat loss and blubber distribution in the beluga Delphinapterus leucas, with comparisons to the narwhal, Monodon monoceros. *Can Bull Fish Aquat Sci* 1990;**224**:129–40.

59. Hokkanen JEI. Temperature regulation of marine mammals. *J Theor Biol* 1990;**145**:465–85.

60. Innes S, Worthy GAJ, Lavigne DM *et al.* Surface area of phocid seals. *Can J Zool* 1990;**68**:2531–8.

61. Lavigne DM, Innes S, Worthy GAJ *et al.* Lower critical temperatures of blue whales, Balaenoptera musculus. *J Theor Biol* 1990;**144**:249–57.

62. Øritsland NA, Markussen NH. Outline of a physiologically based model for population energetics. *Ecol Modell* 1990;**52**:267–88.

63. Ryg M, Lydersen C, Markussen NH *et al.* Estimating the blubber content of phocid seals. *Can J Fish Aquat Sci* 1990;**47**:1223–7.

64. Trites AW. Thermal Budgets and Climate Spaces: The Impact of Weather on the Survival of Galapagos (Arctocephalus galapagoensis Heller) and Northern Fur Seal Pups (Callorhinus ursinus L.). *Funct Ecol* 1990;**4**:753.

65. Worthy GAJ, Edwards EF. Morphometric and biochemical factors affecting heat loss in a small temperate cetacean (Phocoena phocoena) and a small tropical cetacean (Stenella attenuata). *Physiol Zool* 1990;**63**:432–42.

66. Francis JM, Boness DJ. The effect of thermoregulatory behaviour on the mating system of the Juan Fernandez fur seal, Arctocephalus philippii. *Behaviour* 1991;**119**:104–26.

67. Little GJ. Thyroid morphology and function and its role in thermoregulation in the newborn southern elephant seal (Mirounga leonina) at Macquarie Island. *J Anat* 1991;**176**:55–69.

68. Smith TG, Hammill MO, Taugbol G. A review of the developmental, behavioural and physiological adaptations of the ringed seal, Phoca hispida, to life in the Arctic winter. *Arctic* 1991;**44**:124–31.

69. Worthy GAJ. Insulation and thermal balance of fasting harp and grey seal pups. *Comp Biochem Physiol* 1991;**100**:845–51.

70. Cuyler LC, Wiulsrod R, Oritsland NA. Thermal infrared radiation from free living whales. *Mar Mammal Sci* 1992;**8**:120–34.

71. Folkow LP, Blix AS. Metabolic rates of minke whales. *Acta Physiol Scand* 1992;**146**:141–50.

72. Rommel SA, Pabst DA, McLellan WA *et al.* Anatomical evidence for a countercurrent heat exchanger associated with dolphin testes. *Anat Rec* 1992;**232**:150–6.

73. Ponganis PJ, Kooyman GL, Castellini MA *et al.* Muscle temperature and swim velocity profiles during diving in a Weddell seal, Leptonychotes Weddellii. *J Exp Biol* 1993;**183**:341–8.

74. Rommel SA, Pabst D, McLellan WA. Functional Morphology of the Vascular Plexuses Associated With the Cetacean Uterus. *Anat Rec* 1993;**2**.

75. Ryg M, Lydersen C, Knutsen L *et al.* Scaling of insulation in seals and whales. *J Zool* 1993;**230**:193–206.

76. Watts P, Hansen S, Lavigne DM. Models of heat loss by marine mammals: thermoregulation below the zone of irrelevance. *J Theor Biol* 1993;**163**:505–25.

77. Kvadsheim PH, Folkow LP, Blix AS. A new device for measurement of the thermal conductivity of fur and blubber. *J Therm Biol* 1994;**19**:431–5.

78. Rommel SA, Pabst DA, McLellan WA *et al.* Temperature regulation of the testes of the bottlenose dolphin (Tursiops truncatus): evidence from colonic temperatures. *J Comp Physiol B* 1994;**164**:130–4.

79. Beck GG, Smith TG. Distribution of blubber in the northwest Atlantic harp seal, Phoca groenlandica . *Can J Zool* 1995;**73**:1991–8.

80. Boily P. Theoretical Heat Flux in Water and Habitat Selection of Phocid Seals and Beluga Whales during the Annual Molt. *J Theor Biol* 1995;**172**:235–44.

81. Hansen S, Lavigne DM, Innes S. Energy metabolism and thermoregulation in juvenile harbor seals (Phoca vitulina) in air. *Physiol Zool* 1995;**68**:290–315.

82. Kastelein RA, van der Sijs SJ, Staal C *et al.* Blubber thickness in harbour porpoises (Phocoena phocoena). *Food Consumption and Growth in Marine Mammals*. 1995, 179–99.

83. Little GJ. Body temperature in the newborn southern elephant seal, Mirounga leonina, at Macquarie Island. *Mar Mammal Sci* 1995;**11**:480–90.

84. Pabst DA, Rommel SA, McLellan WA *et al.* Thermoregulation of the intra-abdominal testes of the bottlenose dolphin (Tursiops truncatus) during exercise. *J Exp Biol* 1995;**198**:221–6.

85. Rommel SA, Early GA, Matassa KA *et al.* Venous structures associated with thermoregulation of phocid seal reproductive organs. *Anat Rec* 1995;**243**:390–402.

86. Boily P, Lavigne DM. Thermoregulation of juvenile grey seals, Halichoerus grypus, in air. *Can J Zool* 1996;**74**:201–8.

87. Kvadsheim PH, Folkow LP, Blix AS. Thermal conductivity of minke whale blubber. *J Therm Biol* 1996;**21**:123–8.

88. Hansen S, Lavigne DM. Ontogeny of the Thermal Limits in the Harbor Seal (Phoca vitulina). *Physiol Zool* 1997;**70**:85–92.

89. Heyning JE, Mead JG. Thermoregulation in the Mouths of Feeding Gray Whales. *Science (80-)* 1997;**278**:1138–9.

90. Hind AT, Gurney WSC. The metabolic cost of swimming in a marine homeotherm. *J Exp Biol* 1997;**200**:531–42.

91. Kastelein RA, Koene P, Nieuwstraten SH *et al.* Skin surface temperature changes in a harbour porpoise (phocoena phocoena) while on land. *The Biology of the Harbour Porpoise*. 1997, 225–64.

92. Kvadsheim PH, Folkow LP. Blubber and flipper heat transfer in harp seals. *Acta Physiol Scand* 1997;**161**:385–95.

93. Kvadsheim PH, Gotaas ARL, Folkow LP *et al.* An Experimental Validation of Heat Loss Models for Marine Mammals. *J Theor Biol* 1997;**184**:15–23.

94. Rosen DAS, Renouf D. Seasonal Changes in Blubber Distribution in Atlantic Harbor Seals: Indications of Thermodynamic Considerations. *Mar Mammal Sci* 1997;**13**:229–40.

95. Hind AT, Gurney WSC. Are there thermoregulatory constraints on the timing of pupping for harbour seals? *Can J Zool* 1998;**76**:2245–54.

96. Koopman HN. Topographical distribution of the blubber of harbor porpoises (Phocoena phocoena). *J Mammal* 1998;**79**:260–70.

97. Heath ME, Ridgway SH. How dolphins use their blubber to avoid heat stress during encounters with warm water. *Am J Physiol* 1999;**276**:R1188–94.

98. Noren DP, Williams TM, Berry P *et al.* Thermoregulation during swimming and diving in bottlenose dolphins (Tursiops truncatus). *J Comp Physiol B* 1999;**169**:93–9.

99. Williams TM, Noren D, Berry P *et al.* The Diving Physiology of Bottlenose Dolphins (Tursiops Truncatus): III. Thermoregulation at Depth. *J Exp Biol* 1999;**202**:2763–9.

100. Boily P, Kvadsheim PH, Folkow LP. Cutaneous Heat Flux Models do not Reliably Predict Metabolic Rates of Marine Mammals. *J Theor Biol* 2000;**207**:317–23.

101. Boyd IL. Skin temperatures during free-ranging swimming and diving in Antarctic fur seals. *J Exp Biol* 2000;**203**:1907–14.

102. Donohue MJ, Costa DP, Goebel ME *et al.* The ontogeny of metabolic rate and thermoregulatory capabilities of northern fur seal, Callorhinus ursinus, pups in air and water. *J Exp Biol* 2000;**203**:1003–16.

103. Mauck B, Eysel U, Dehnhardt G. Selective heating of vibrissal follicles. *J Exp Biol* 2000;**203**:2125–31.

104. Heyning JE. Thermoregulation in feeding baleen whales: Morphological and physiological evidence. *Aquat Mamm* 2001;**27**:284–8.

105. Jobsis PD, Ponganis PJ, Kooyman GL. Effects of training on forced submersion responses in harbor seals. *J Exp Biol* 2001;**204**:3877–85.

106. Rommel SA, Pabst DA, McLellan WA. Functional morphology of venous structures associated with the male and female reproductive systems in Florida manatees (Trichechus manatus latirostris). *Anat Rec* 2001;**264**:339–47.

107. Koopman HN, Pabst DA, McLellan WA *et al.* Changes in blubber distribution and morphology associated with starvation in the harbor porpoise (Phocoena phocoena): Evidence for regional differences in blubber structure and function. *Physiol Biochem Zool* 2002;**75**:498–512.

108. Kvadsheim PH, Aarseth JJ. Thermal function of phocid seal fur. *Mar Mammal Sci* 2002;**18**:952–62.

109. Meagher EM, McLellan WA, Westgate AJ *et al.* The relationship between heat flow and vasculature in the dorsal fin of wild bottlenose dolphins Tursiops truncatus. *J Exp Biol* 2002;**205**:3475–86.

110. Noren DP. Thermoregulation of Weaned Northern Elephant Seal (Mirounga angustirostris) Pups in Air and Water. *Physiol Biochem Zool* 2002;**75**:513–23.

111. Twiss SD, Wright NC, Dunstone N *et al.* Behavioral evidence of thermal stress from overheating in UK breeding gray seals. *Mar Mammal Sci* 2002;**18**:455–68.

112. Mauck B, Bilgmann K, Jones DD *et al.* Thermal windows on the trunk of hauled-out seals: hot spots for thermoregulatory evaporation? *J Exp Biol* 2003;**206**:1727–38.

113. Rommel SA, Caplan H. Vascular adaptations for heat conservation in the tail of Florida manatees (Trichechus manatus latirostris). *J Anat* 2003;**202**:343–53.

114. Rosen DAS, Trites AW. No Evidence for Bioenergetic Interaction between Digestion and Thermoregulation in Steller Sea Lions Eumetopias jubatus. *Physiol Biochem Zool* 2003;**76**:899–906.

115. Elsner R, Meiselman HJ, Baskurt OK. Temperature-viscosity relations of bowhead whale blood: A possible mechanism for maintaining cold blood flow. *Mar Mammal Sci* 2004;**20**:339–44.

116. Elsner R, George JC, O'Hara T. Vasomotor responses of isolated peripheral blood vessels from bowhead whales: Thermoregulatory implications. *Mar Mammal Sci* 2004;**20**:546–53.

117. Ortiz RM, Worthy GAJ. Could lower body fat mass contribute to cold-water susceptibility in calves of the West Indian manatee (Trichechus manatus)? *Mar Mammal Sci* 2004;**20**:176–83.

118. Rutishauser MR, Costa DP, Goebel ME *et al.* Ecological implications of body composition and thermal capabilities in young antarctic fur seals (Arctocephalus gazella). *Physiol Biochem Zool* 2004;**77**:669–81.

119. Samuel AM, Worthy GAJ. Variability in fatty acid composition of bottlenose dolphin (Tursiops truncatus) blubber as a function of body site, season, and reproductive state. *Can J Zool* 2004;**82**:1933–42.

120. Silva RG. Assessment of body surface temperature in cetaceans: an iterative approach. *Brazilian J Biol* 2004;**64**:719–24.

121. Struntz DJ, McLellan WA, Dillaman RM *et al.* Blubber Development in Bottlenose Dolphins (Tursiops truncatus). *J Morphol* 2004;**259**:7–20.

122. Bossart GD, Meisner RA, Rommel SA *et al.* Pathological features of the Florida manatee cold stress syndrome. *Aquat Mamm* 2005;**29**:9–17.

123. Dunkin RC, McLellan WA, Blum JE *et al.* The ontogenetic changes in the thermal properties of blubber from Atlantic bottlenose dolphin Tursiops truncatus. *J Exp Biol* 2005;**208**:1469–80.

124. Harding KC, Fujiwara M, Axberg Y *et al.* Mass-dependent energetics and survival in Harbour Seal pups. *Funct Ecol* 2005;**19**:129–35.

125. Kvadsheim PH, Folkow LP, Blix AS. Inhibition of shivering in hypothermic seals during diving. *Am J Physiol Regul Integr Comp Physiol* 2005;**289**:326–31.

126. McCafferty DJ, Moss S, Bennett K *et al.* Factors influencing the radiative surface temperature of grey seal (Halichoerus grypus) pups during early and late lactation. *J Comp Physiol B Biochem Syst Environ Physiol* 2005;**175**:423–31.

127. Rotherham LS, van der Merwe M, Bester MN *et al.* Morphology and distribution of sweat glands in the Cape fur seal, Arctocephalus pusillus pusillus (Carnivora:Otariidae). *Aust J Zool* 2005;**53**:295–300.

128. Thornton SJ, Hochachka PW, Crocker DE *et al.* Stroke volume and cardiac output in juvenile elephant seals during forced dives. *J Exp Biol* 2005;**208**:3637–43.

129. Willis K, Horning M. A novel approach to measuring heat flux in swimming animals. *J Exp Mar Bio Ecol* 2005;**315**:147–62.

130. Willis K, Horning M, Rosen DAS *et al.* Spatial variation of heat flux in Steller sea lions: evidence for consistent avenues of heat exchange along the body trunk. *J Exp Mar Bio Ecol* 2005;**315**:163–75.

131. Beentjes MP. Behavioral thermoregulation of the New Zealand sea lion (Phocarctos hookeri). *Mar Mammal Sci* 2006;**22**:311–25.

132. Ponganis PJ, Stockard TK, Levenson DH *et al.* Cardiac output and muscle blood flow during rest-associated apneas of elephant seals. *Comp Biochem Physiol - A Mol Integr Physiol* 2006;**144**:105–11.

133. Koopman HN. Phylogenetic, ecological, and ontogenetic factors influencing the biochemical structure of the blubber of odontocetes. *Mar Biol* 2007;**151**:277–91.

134. Mellish J-AE, Horning M, York AE. Seasonal and Spatial Blubber Depth Changes in Captive Harbor Seals (Phoca vitulina) and Steller's Sea Lions (Eumetopias jubatus). *J Mammal* 2007;**88**:408–14.

135. Werth AJ. Adaptations of the cetacean hyolingual apparatus for aquatic feeding and thermoregulation. *Anat Rec* 2007;**290**:546–68.

136. Westgate AJ, McLellan WA, Wells RS *et al.* A new device to remotely measure heat flux and skin temperature from free-swimming dolphins. *J Exp Mar Bio Ecol* 2007;**346**:45–59.

137. Hasselberg BA, Thomas JA. Using an Infrared Temperature Sensor to Study Microhabitat Selection in Captive California Sea Lions (Zalophus californianus). *Aquat Mamm* 2008;**34**:212–28.

138. Meagher EM, McLellan WA, Westgate AJ *et al.* Seasonal patterns of heat loss in wild bottlenose dolphins (Tursiops truncatus). *J Comp Physiol B* 2008;**178**:529–43.

139. Montie EW, Garvin SR, Fair PA *et al.* Blubber morphology in wild bottlenose dolphins (Tursiops truncatus) from the Southeastern United States: Influence of geographic location, age class, and reproductive state. *J Morphol* 2008;**269**:496–511.

140. Noren SR, Pearson LE, Davis J *et al.* Different thermoregulatory strategies in nearly weaned pup, yearling, and adult Weddell seals (Leptonychotes weddelli). *Physiol Biochem Zool* 2008;**81**:868–79.

141. Ponganis PJ, Kreutzer U, Stockard TK *et al.* Blood flow and metabolic regulation in seal muscle during apnea. *J Exp Biol* 2008;**211**:3323–32.

142. Yeates LC, Houser DS. Thermal tolerance in bottlenose dolphins (Tursiops truncatus). *J Exp Biol* 2008;**211**:3249–57.

143. Castellini MA, Trumble SJ, Mau TL *et al.* Body and blubber relationships in Antarctic pack ice seals: Implications for blubber depth patterns. *Physiol Biochem Zool* 2009;**82**:113–20.

144. Hammill MO, Ryg M, Chabot D. Seasonal changes in energy requirements of harp seals. *J Northwest Atl Fish Sci* 2009;**42**:135–52.

145. Liwanag HEM, Williams TM, Costa DP *et al.* The effects of water temperature on the energetic costs of juvenile and adult California sea lions (Zalophus californianus): the importance of skeletal muscle thermogenesis for thermal balance. *J Exp Biol* 2009;**212**:3977–84.

146. Noren SR, Wells RS. Blubber Deposition during Ontogeny in Free-Ranging Bottlenose Dolphins: Balancing Disparate Roles of Insulation and Locomotion. *J Mammal* 2009;**90**:629–37.

147. Barbieri MM, McLellan WA, Wells RS *et al.* Using infrared thermography to assess seasonal trends in dorsal fin surface temperatures of free-swimming bottlenose dolphins (Tursiops truncatus) in Sarasota Bay, Florida. *Mar Mammal Sci* 2010;**26**:53–66.

148. Blix AS, Walløe L, Messelt EB *et al.* Selective brain cooling and its vascular basis in diving seals. *J Exp Biol* 2010;**213**:2610–6.

149. Liwanag HEM. Energetic Costs and Thermoregulation in Northern Fur Seal (Callorhinus ursinus) Pups: The Importance of Behavioral Strategies for Thermal Balance in Furred Marine Mammals. *Physiol Biochem Zool* 2010;**83**:898–910.

150. Meir JU, Ponganis PJ. Blood Temperature Profiles of Diving Elephant Seals. *Physiol Biochem Zool* 2010;**83**:531–40.

151. Nienaber J, Thomton J, Horning M *et al.* Surface temperature patterns in seals and sea lions: A validation of temporal and spatial consistency. *J Therm Biol* 2010;**35**:435–40.

152. Norris AL, Houser DS, Crocker DE. Environment and activity affect skin temperature in breeding adult male elephant seals (Mirounga angustirostris). *J Exp Biol* 2010;**213**:4205–12.

153. Houser DS, Yeates LC, Crocker DE. Cold stress induces an adrenocortical response in bottlenose dolphins (Tursiops truncatus). *J Zoo Wildl Med* 2011;**42**:565–71.

154. Bagge LE, Koopman HN, Rommel SA *et al.* Lipid class and depth-specific thermal properties in the blubber of the short-finned pilot whale and the pygmy sperm whale. *J Exp Biol* 2012;**215**:4330–9.

155. Durban JW, Pitman RL. Antarctic killer whales make rapid, round-trip movements to subtropical waters: evidence for physiological maintenance migrations? *Biol Lett* 2012;**8**:274–7.

156. Erdsack N, Hanke FD, Dehnhardt G *et al.* Control and amount of heat dissipation through thermal windows in harbor seals (Phoca vitulina). *J Therm Biol* 2012;**37**:537–44.

157. Khamas WA, Smodlaka H, Leach-Robinson J *et al.* Skin histology and its role in heat dissipation in three pinniped species. *Acta Vet Scand* 2012;**54**:46.

158. Liwanag HEM, Berta A, Costa DP *et al.* Morphological and thermal properties of mammalian insulation: the evolution of fur for aquatic living. *Biol J Linn Soc* 2012;**106**:926–39.

159. Liwanag HEM, Berta A, Costa DP *et al.* Morphological and thermal properties of mammalian insulation: the evolutionary transition to blubber in pinnipeds. *Biol J Linn Soc* 2012;**107**:774–87.

160. McClelland SJ, Gay M, Ann D *et al.* Microvascular Patterns in the Blubber of Shallow and Deep Diving Odontocetes. *J Morphol* 2012;**273**:932–42.

161. Mellish J, Nienaber J, Polasek L *et al.* Beneath the surface: Profiling blubber depth in pinnipeds with infrared imaging. *J Therm Biol* 2012;**38**:10–3.

162. Paterson W, Sparling CE, Thompson D *et al.* Seals like it hot: Changes in surface temperature of harbour seals (Phoca vitulina) from late pregnancy to moult. *J Therm Biol* 2012;**37**:454–61.

163. Erdsack N, Dehnhardt G, Hanke W. Coping with Heat: Function of The Natal Coat of Cape Fur Seal (Arctocephalus Pusillus Pusillus) Pups in Maintaining Core Body Temperature. *PLoS One* 2013;**8**:72081.

164. Ford TJ, Werth AJ, George JC. An Intraoral Thermoregulatory Organ in the Bowhead Whale (Balaena mysticetus), the Corpus Cavernosum Maxillaris. *Anat* 2013;**296**:701–8.

165. Rodríguez-Prieto V, Rubio-García A, Melero M *et al.* Identification of the pattern of appearance and development of thermal windows in the skin of juvenile Pacific walruses (Odobenus rosmarus divergens) in a controlled environment. *Mar Mammal Sci* 2013;**29**:167–76.

166. Dalton AJM, Rosen DAS, Trites AW. Broad thermal capacity facilitates the primarily pelagic existence of northern fur seals (Callorhinus ursinus). *Mar Mammal Sci* 2014;**30**:994–1013.

167. Erdsack N, Dehnhardt G, Hanke W. Thermoregulation of the vibrissal system in harbor seals (Phoca vitulina) and Cape fur seals (Arctocephalus pusillus pusillus). *J Exp Mar Bio Ecol* 2014;**452**:111–8.

168. Garlepp L, Logan M, Kirkwood R. Behavioral responses of Australian fur seals (Arctocephalus pusillus doriferus) to environmental variations. *Mar Mammal Sci* 2014;**30**:978–93.

169. Horgan P, Booth D, Nichols C *et al.* Insulative capacity of the integument of the dugong (Dugong dugon): thermal conductivity, conductance and resistance measured by in vitro heat flux. *Mar Biol* 2014;**161**:1395–407.

170. Liwanag HEM, Oraze J, Costa DP *et al.* Thermal benefits of aggregation in a large marine endotherm: Huddling in California sea lions. *J Zool* 2014;**293**:152–9.

171. Pearson LE, Liwanag HEM, Hammill MO *et al.* Shifts in thermoregulatory strategy during ontogeny in harp seals (Pagophilus groenlandicus). *J Therm Biol* 2014;**44**:93–102.

172. Pearson LE, Liwanag HEM, Hammill MO *et al.* To each its own: Thermoregulatory strategy varies among neonatal polar phocids. *Comp Biochem Physiol -Part A Mol Integr Physiol* 2014;**178**:59–67.

173. Rosen DAS, Trites AW. Thermal limits in young northern fur seals, Callorhinus ursinus. *Mar Mammal Sci* 2014;**30**:1014–28.

174. Ball HC, Stavarz M, Oldaker J *et al.* Seasonal and Ontogenetic Variation in Subcutaneous Adipose Of the Bowhead Whale (Balaena mysticetus). *Anat Rec* 2015;**298**:1416–23.

175. Ekdale EG, Kienle SS. Passive Restriction of Blood Flow and Counter-Current Heat Exchange Via Lingual Retia in the Tongue of a Neonatal Gray Whale Eschrichtius robustus (Cetacea, Mysticeti). *Anat Rec* 2015;**298**:675–9.

176. Erdsack N, Dehnhardt G, Witt M *et al.* Unique fur and skin structure in harbor seals (Phoca vitulina)-thermal insulation, drag reduction, or both? *J R Soc Interface* 2015;**12**:1–7.

177. Gmuca N V., Pearson LE, Burns JM *et al.* The fat and the furriest: Morphological changes in harp seal fur with ontogeny. *Physiol Biochem Zool* 2015;**88**:158–66.

178. Gómez-Campos E, Borrell A, Correas J *et al.* Topographical variation in lipid content and morphological structure of the blubber in the striped dolphin. *Sci Mar* 2015;**79**:189–97.

179. Hashimoto O, Ohtsuki H, Kakizaki T *et al.* Brown Adipose Tissue in Cetacean Blubber. *PLoS One* 2015;**10**, DOI: 10.1371/journal.pone.0116734.

180. Hindle AG, Horning M, Mellish JAE. Estimating total body heat dissipation in air and water from skin surface heat flux telemetry in Weddell seals. *Anim Biotelemetry* 2015;**3**:1–11.

181. Melero M, Rodríguez-Prieto V, Rubio-García A *et al.* Thermal reference points as an index for monitoring body temperature in marine mammals. *BMC Res Notes* 2015;**8**:411.

182. Mellish J-A, Hindle A, Skinner J *et al.* Heat loss in air of an Antarctic marine mammal, the Weddell seal. *J Comp Physiol B* 2015;**185**:143–52.

183. Noren SR, Udevitz MS, Triggs L *et al.* Identifying a reliable blubber measurement site to assess body condition in a marine mammal with topographically variable blubber, the Pacific walrus. *Mar Mammal Sci* 2015;**31**:658–76.

184. Sakurai Y, Okamatsu-Ogura Y, Saito M *et al.* Brown adipose tissue expresses uncoupling protein 1 in newborn harbor seals (Phoca vitulina). *Mar Mammal Sci* 2015;**31**:818–27.

185. Zeng X, Ji J, Hao Y *et al.* Topographical distribution of blubber in finless porpoises (neophocaena asiaeorientalis sunameri): A result from adapting to living in coastal waters. *Zool Stud* 2015;**54**:1–11.

186. Codde SA, Allen SG, Houser DS *et al.* Effects of environmental variables on surface temperature of breeding adult female northern elephant seals, Mirounga angustirostris, and pups. *J Therm Biol* 2016;**61**:98–105.

187. Cornick LA, Quakenbush LT, Norman SA *et al.* Seasonal and developmental differences in blubber stores of beluga whales in Bristol Bay, Alaska using high-resolution ultrasound. *J Mammal* 2016;**97**:1238–48.

188. Beltran RS, Testa JW, Burns JM. An agent-based bioenergetics model for predicting impacts of environmental change on a top marine predator, the Weddell seal. *Ecol Modell* 2017;**351**:36–50.

189. Ladds MA, Slip DJ, Harcourt RG *et al.* Intrinsic and extrinsic influences on standard metabolic rates of three species of Australian otariid. *Conserv Biol* 2017;**5**:1–14.

190. Sharma N, Liwanag HEM. The effects of submergence on the thermal function of pinniped fur. *Mar Mammal Sci* 2017;**33**:611–20.

191. Singleton EM, McLellan WA, Koopman HN *et al.* Lipid composition and thermal properties of the blubber of Gervais' beaked whale (Mesoplodon europaeus) across ontogeny. *Mar Mammal Sci* 2017;**33**:695–705.

192. Chaise LL, Prinet I, Toscani C *et al.* Local weather and body condition influence habitat use and movements on land of molting female southern elephant seals (Mirounga leonina). *Ecol Evol* 2018;**8**:6081–90.

193. Erdsack N, McCully Phillips SR, Rommel SA *et al.* Heat flux in manatees: an individual matter and a novel approach to assess and monitor the thermal state of Florida manatees (Trichechus manatus latirostris). *J Comp Physiol B* 2018;**188**:717–27.

194. García-Aguilar MC, Turrent C, Elorriaga-Verplancken FR *et al.* Climate change and the northern elephant seal (Mirounga angustirostris) population in Baja California, Mexico. *PLoS One* 2018;**13**:1–15.

195. Krmpotic CM, Loza CM, Negrete J *et al.* Integument in Antarctic seals: A comparative study and its relation to extreme environments. *Acta Zool* 2018;**99**:281–95.

196. Rojano-Donãte L, McDonald BI, Wisniewska DM *et al.* High field metabolic rates of wild harbour porpoises. *J Exp Biol* 2018;**221**, DOI: 10.1242/jeb.185827.

197. Zeh DR, Heupel MR, Hamann M *et al.* Evidence of behavioural thermoregulation by dugongs at the high latitude limit to their range in eastern Australia. *J Exp Mar Bio Ecol* 2018;**508**:27–34.

198. Adamczak SK, Pabst A, McLellan WA *et al.* Using 3D Models to Improve Estimates of Marine Mammal Size and External Morphology. *Front Mar Sci* 2019;**6**:1–12.

199. Chaise LL, McCafferty DJ, Krellenstein A *et al.* Environmental and physiological determinants of huddling behavior of molting female southern elephant seals (Mirounga leonina). *Physiol Behav* 2019;**199**:182–90.

200. Guerrero AI, Rogers TL. From low to high latitudes: changes in fatty acid desaturation in mammalian fat tissue suggest a thermoregulatory role. *BMC Evol Biol* 2019;**19**:1–12.

201. Ji J, Nabi G, Zeng X *et al.* Histological variation in blubber morphology of the endangered east asian finless porpoise (Neophocaena asiaeorientalis sunameri) with ontogeny and reproductive states. *Zool Stud* 2019;**58**:1–11.

202. Kastelein RA, Helder-Hoek L, Jennings N *et al.* Reduction in body mass and blubber thickness of harbor porpoises (Phocoena phocoena) due to near-fasting for 24 hours in four seasons. *Aquat*

Mamm 2019;**45**:37–47.

203. Lonati GL, Singleton EM, Phelps CE *et al.* The density of odontocete integument depends on blubber lipid composition and temperature. *Mar Mammal Sci* 2019;**35**:595–616.

204. Pearson LE, Weitzner EL, Burns JM *et al.* From ice to ocean: changes in the thermal function of harp seal pelt with ontogeny. *J Comp Physiol B* 2019;**189**:501–11.

205. Pitman RL, Durban JW, Joyce T *et al.* Skin in the game: Epidermal molt as a driver of long-distance migration in whales. *Mar Mammal Sci* 2019:1–30.

206. Plön S, Frainer G, Wedderburn-Maxwell A *et al.* Dorsal fin and hump vascular anatomy in the Indo-Pacific humpback dolphin (Sousa plumbea) and the Indo-Pacific bottlenose dolphin (Tursiops aduncus). *Mar Mammal Sci* 2019;**35**:684–95.

207. Tuneu-Corral C, Szteren D, Cassini MH. Living on the edge: thermoregulatory behaviour of South American sea lions, Otaria flavescens, at the northern limit of their Atlantic distribution. *Acta Ethol* 2019;**22**:67–71.

208. Adamczak SK, Ann Pabst D, McLellan WA *et al.* Do bigger bodies require bigger radiators? Insights into thermal ecology from closely related marine mammal species and implications for ecogeographic rules. *J Biogeogr* 2020;**47**:1193–206.

209. Adamczak SK, McLellan WA, Read AJ *et al.* The impact of temperature at depth on estimates of thermal habitat for short-finned pilot whales. *Mar Mammal Sci* 2020:1–14.

210. Chambault P, Tervo OM, Garde E *et al.* The impact of rising sea temperatures on an Arctic top predator, the narwhal. *Sci Rep* 2020;**10**:1–10.

211. da Silva AP, Machado ASD, Le Bas AE *et al.* The skin structures and their role in the thermoregulation of the South American fur seal (Arctocephalus australis). *Anat Rec* 2020;**303**:3155–67.

212. Haase CG, Fletcher RJ, Slone DH *et al.* Traveling to thermal refuges during stressful temperatures leads to foraging constraints in a central-place forager. *J Mammal* 2020;**101**:271–80.

213. Heide-Jørgensen MP, Blackwell SB, Williams TM *et al.* Some like it cold: Temperature-dependent habitat selection by narwhals. *Ecol Evol* 2020;**10**:8073–90.

214. Walcott SM, Kirkham AL, Burns JM. Thermoregulatory costs in molting Antarctic Weddell seals: impacts of physiological and environmental conditions. *Conserv Physiol* 2020;**8**:1–14.

215. Guerrero AI, Rogers TL, Sepúlveda M *et al.* Conditions influencing the appearance of thermal windows and the distribution of surface temperature in hauled-out southern elephant seals. *Conserv Physiol* 2021;**9**, DOI: 10.1093/conphys/coaa141.

216. John JS, Thometz NM, Boerner K *et al.* Metabolic trade-offs in tropical and subtropical marine mammals: unique maintenance and locomotion costs in West Indian manatees and Hawaiian monk seals. *J Exp Biol* 2021;**224**, DOI: 10.1242/jeb.237628.

217. Sumich JL. Why Baja? A bioenergetic model for comparing metabolic rates and thermoregulatory costs of gray whale calves (Eschrichtius robustus). *Mar Mammal Sci* 2021;**37**:870–87.

218. Wright T, Davis RW, Pearson HC *et al.* Skeletal muscle thermogenesis enables aquatic life in the smallest marine mammal. *Science (80-)* 2021;**373**:223–5.
